# Ectodysplasin target gene Fgf20 regulates mammary bud growth and ductal invasion and branching during puberty

**DOI:** 10.1038/s41598-017-04637-1

**Published:** 2017-07-11

**Authors:** Teresa Elo, Päivi H. Lindfors, Qiang Lan, Maria Voutilainen, Ewelina Trela, Claes Ohlsson, Sung-Ho Huh, David M. Ornitz, Matti Poutanen, Beatrice A. Howard, Marja L. Mikkola

**Affiliations:** 1 Developmental Biology Program, Institute of Biotechnology, University of Helsinki, Helsinki, Finland; 2 Center for Bone and Arthritis Research, Department of Internal Medicine, Institute of Medicine, Sahlgrenska Academy, University of Gothenburg, Gothenburg, Sweden; 3 Department of Developmental Biology, Washington University School of Medicine, St. Louis, Missouri USA; 4 Department of Physiology and Turku Center for Disease Modeling, Institute of Biomedicine, University of Turku, Turku, Finland; 5 The Breast Cancer Now Toby Robins Research Centre, Division of Breast Cancer, the Institute of Cancer Research, London, United Kingdom; 6 Holland Regenerative Medicine Program and Department of Developmental Neuroscience, Munroe-Meyer Institute, University of Nebraska Medical Center, Omaha, Nebraska USA

## Abstract

Mammary gland development begins with the appearance of epithelial placodes that invaginate, sprout, and branch to form small arborized trees by birth. The second phase of ductal growth and branching is driven by the highly invasive structures called terminal end buds (TEBs) that form at ductal tips at the onset of puberty. Ectodysplasin (Eda), a tumor necrosis factor-like ligand, is essential for the development of skin appendages including the breast. In mice, Eda regulates mammary placode formation and branching morphogenesis, but the underlying molecular mechanisms are poorly understood. Fibroblast growth factor (Fgf) receptors have a recognized role in mammary ductal development and stem cell maintenance, but the ligands involved are ill-defined. Here we report that Fgf20 is expressed in embryonic mammary glands and is regulated by the Eda pathway. Fgf20 deficiency does not impede mammary gland induction, but compromises mammary bud growth, as well as TEB formation, ductal outgrowth and branching during puberty. We further show that loss of *Fgf20* delays formation of Eda-induced supernumerary mammary buds and normalizes the embryonic and postnatal hyperbranching phenotype of Eda overexpressing mice. These findings identify a hitherto unknown function for Fgf20 in mammary budding and branching morphogenesis.

## Introduction

Mammalian fibroblast growth factors (Fgfs) constitute a family of 18 secreted polypeptide growth factors with diverse roles in multiple developmental processes^1^. Secreted Fgfs serve as ligands for single-pass transmembrane receptor tyrosine kinases (Fgfr1–4). With the exception of Fgfr4, alternative splicing of Fgfrs produces two isoforms (IIIb and IIIc isoforms) with different ligand binding specificities. In general, mesenchymal Fgfs encage epithelial IIIb receptor isoforms, whereas epithelial Fgfs signal to mesenchymal IIIc receptors^2^. Activation of any of these isoforms can trigger several signalling cascades including the RAS-MAPK, PI3K, STAT, and PLCγ pathways leading to various cellular responses in a context dependent manner. The fact that all Fgfs can bind to several Fgfrs, and vice versa, produces a high degree of redundancy within the system^1^.

Mammary gland development proceeds via distinct stages: the hormone-independent embryonic and prepubertal morphogenesis, and the subsequent pubertal, pregnancy, lactation, and involution stages driven by hormonal cues^3^. In mice, mammary gland development commences at ~embryonic day 11 (E11) with the sequential appearance of five pairs of mammary primordia called placodes^4^. Placodes are local epithelial thickenings that gradually invaginate to the underlying tissue to form buds, which from E12.5 onwards are surrounded by a specialized condensed mammary mesenchyme. Mammary buds grow relatively slowly in size until E15–E16 when a primary sprout forms. The sprout invades the secondary mammary mesenchyme, the precursor of the fatty adult stroma, and branching morphogenesis begins. By birth (E19–E20), a small ductal tree with 10–15 branches has formed. Nipple sheath, as well as lumen formation also start at late embryogenesis^4^.

Postnatal growth and branching is relatively slow until the onset of puberty, which begins at ~3 week of age as a response to onset of ovarian steroid hormone production and is associated with remarkable morphological changes. Bulbous epithelial structures called terminal end buds (TEBs) form at the tips of mammary ducts and start invading into the fat pad^3^. This phase of rapid growth, which includes extensive ductal elongation, branching, and extracellular matrix (ECM) degradation, continues until the ductal network reaches the borders of the fat pad and the TEBs regress by the age of 10–12 weeks^5^.

Mammary gland morphogenesis relies on sequential and reciprocal crosstalk between the epithelium and the underlying stroma and these tissue interactions guide all aspects of mammary gland development from induction to involution^6^. This crosstalk is mediated by conserved signaling pathways, of which the Wnt and Fgf pathways are the most critical ones during the early stages of mammogenesis^7, 8^. Loss of the Wnt signal mediator *Lef1* leads to absence of placodes 2 and 3^9^, whereas epithelial overexpression of the soluble Wnt inhibitor Dkk1 prevents mammary placode formation altogether^10^. Deficiency in *Fgf10* or its receptor *Fgfr2b* blocks induction of all mammary placodes except the fourth^11^. The initiation of bud outgrowth is triggered by epithelially expressed parathyroid hormone related peptide (PTHrP, a.k.a. parathyroid hormone like hormone): mice null for *PTHrP* or its mesenchymal receptor (*Pth1r*) display little to no bud sprouting^12^. Disruption of canonical Wnt signaling pathway components, such as Lrp6 results in stunted embryonic branching morphogenesis and underdeveloped fat pad^13^. Ductal growth and branching is also compromised, albeit to a lesser extent, in epidermal growth factor receptor (*Egfr*) null neonates^14^.

Pubertal branching morphogenesis is regulated by systemic hormones, especially estradiol and growth hormone (GH)^3, 15^. A critical factor induced by estrogen receptor α (Esr1) in the mammary epithelium is the Egf family member amphiregulin (Areg), which activates stromal Egfr signaling^16–18^. Accordingly, *Areg* and *Esr1* knockout mice display a highly similar pubertal phenotype characterized by failure in TEB formation and ductal elongation^18–20^. GH signalling is essential in the mammary stroma where its effects are mediated by insulin-like growth factor 1 (Igf-1), which in turn promotes epithelial cell proliferation and survival^3^. Both GH receptor and *Igf-1* null mice exhibit greatly limited ductal outgrowth^21, 22^. In addition, several other signaling molecules regulate ductal morphogenesis during puberty although their link to hormone signaling is less clear^3, 23^.

Also Fgf signaling plays an important role in mammary branching morphogenesis, as well as in maintaining homeostasis in the adult. Thus far, functional studies have focused on epithelial Fgfrs, in particular Fgfr2b, or their stromal ligands. The single pair (bud 4) of *Fgf10* null mammary primordia sprouts, but shows either absent or very rudimentary ramifications^11^. Several studies have demonstrated the crucial role of Fgfr2 signaling in postnatal development including induction and maintenance of the TEBs, and in ability of mammary stem cells to repopulate the fat pad in transplantation assays^24–27^. Furthermore, conditional epithelial deletion of Fgfr1 leads to a ductal outgrowth phenotype, albeit transient, which is evident already at the onset of puberty^26^.

Ectodysplasin-A1 (hereafter Eda), a member of the tumor necrosis factor (Tnf) superfamily has recently emerged as an important regulator of mammary gland development. Eda signaling is mediated via its receptor Edar and culminates in the activation of the transcription factor NF-κB^28^. Eda pathway loss- and gain-of-function mouse models have been highly informative in elucidating the role of this pathway in mammary gland biology^29–31^. Eda is dispensable for mammary placode formation, yet Eda-overexpressing (K14-*Eda*) mice develop supernumerary mammary glands along and anterior to the milk line^29, 32, 33^. Deficiency in Eda, Edar, or NF-κB leads to smaller ductal trees, a phenotype that manifests from embryogenesis up to at least 6 weeks of age. The converse is observed in Eda and Edar overexpressing mice^30, 34^. In humans, inactivating mutations in the Eda pathway genes cause a syndrome characterized by defective development of several ectodermal organs including the breast^35, 36^. In order to identify the transcriptional mediators of Eda/Edar/NF-κB, we performed microarray profiling of embryonic *Eda* null mammary buds after a 4-hour *ex vivo* exposure to control medium or recombinant Eda protein. This screen revealed several putative Eda target genes including *Fgf20* ^33^, a member of the Fgf9 subfamily comprising of Fgf9, Fgf16, and Fgf20^1^. Our previous studies have identified Fgf20 as an important downstream effector of Eda in developing hair follicles and teeth^37–39^. The present study unveiled an important role for Fgf20 in mammogenesis.

## Results

### Fgf20 is expressed in the embryonic mammary buds

**Figure 1:**
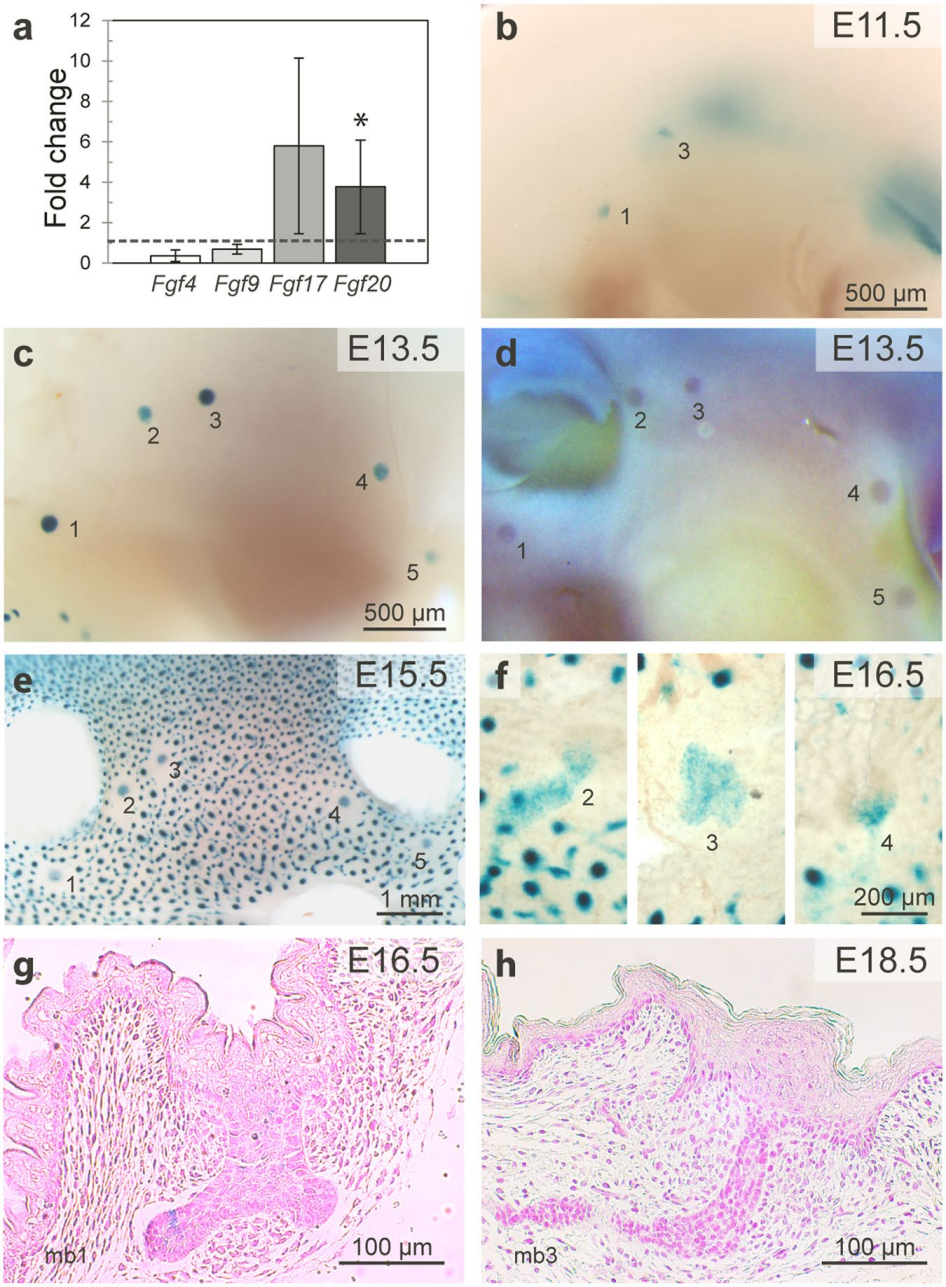
Fgf20 is induced by Eda and is expressed in embryonic mammary glands. (**a**) qRT-PCR analysis of *Fgf4* (n = 4), *Fgf 9* (n = 4), *Fgf17* (n = 6) and *Fgf20* (n = 7) expression in E13.5 *Eda* ^−/−^ mammary buds after 4 h treatment with Eda protein *ex vivo*. Values represent mean ± SD. (**b**,**c**) X-gal*-*stained whole mounts of *Fgf20* ^*LacZ*/+^ embryos at E11.5 (**b**) and E13.5 (**c**) showing positive staining in the developing mammary buds (numbered). (**d**) *In situ* hybridization of a WT embryo with an *Fgf20* specific probe at E13.5. (**e**,**f**) X-Gal stained whole mount of E15.5 whole embryo (**e**) and dissected skin of E16.5 embryo (**f**) showing staining in the developing mammary buds (numbered) and hair follicles. (**g**,**h**) Representative figures of histological sections of X-Gal whole mount-stained mammary glands of *Fgf20* ^*LacZ*/+^ embryos at E16.5 (**g**) and E18.5 (**h**). *p < 0.05. At least two litters of *Fgf20* ^*LacZ*/+^ embryos per stage were analyzed. *p < 0.05. mb, mammary bud.

We have previously shown by microarray profiling that a short treatment with recombinant Eda protein upregulates the expression of *Fgf17* and *Fgf20* in the mammary buds of E13.5 *Eda*−/− embryos *ex vivo* ^33^. Quantitative RT-PCR was used to validate these findings, as well as the expression of *Fgf4* and *Fgf9*, two Fgf family members reported to be expressed in the mammary buds^40^ but not upregulated by Eda in the microarray. In line with the microarray results, after 4 hours Eda-treatment, of these only *Fgf17* and *Fgf20* were upregulated 5.8-fold (p = 0.042) and 3.8-fold (p = 0.019), respectively (Fig. 1a). However, analysis of the absolute mRNA quantity indicated that *Fgf17* is expressed at a very low level, and thus the role of Fgf17 in mammary gland development was not analyzed further.

In order to analyze expression of *Fgf20* in embryonic mammary glands, we took advantage of the *Fgf20-LacZ* knock-in allele^41^ and performed X-gal staining on *Fgf20* ^*LacZ*/+^ embryos between E10.5 and E18.5. Expression of *Fgf20-LacZ* was detected earliest at ~E11.25 in the mammary bud 1 (data not shown), and at E11.5 in the buds 1 and 3 (Fig. 1b). At E13.5, *Fgf20-LacZ* expression was detected in all mammary buds (Fig. 1b) and accordingly, *in situ* hybridization with an *Fgf20* specific probe showed positive signal in wildtype embryos at the same stage (Fig. 1d). The *Fgf20-LacZ* expression was still relatively strong in the mammary buds at E15.5 (Fig. 1e) but was substantially downregulated at E16.5 (Fig. 1f,g). At E18.5, no expression of *Fgf20-LacZ* could be detected in the mammary glands by X-gal staining (Fig. 1h) or immunohistochemical staining with anti-β-galactosidase antibody, although expression in hair follicles was readily observed (Fig. S1a), as reported previously^39^. At postnatal stages, expression of *Fgf20-LacZ* was assessed by X-gal staining and anti-β-galactosidase antibody in mammary glands of 3-, 5- and 7-week-old *Fgf20* ^*LacZ*/+^ and *Fgf20* ^*LacZ/LacZ*^ mice, and by qRT-PCR in samples from 3 different regions (proximal to nipple, middle, and distal to nipple) of 5-week old glands. No expression was detected in the postnatal mammary gland by any of the methods used (Supplementary Fig. S1).

### Eda levels influence the expression of Fgf20 *in vivo*

**Figure 2:**
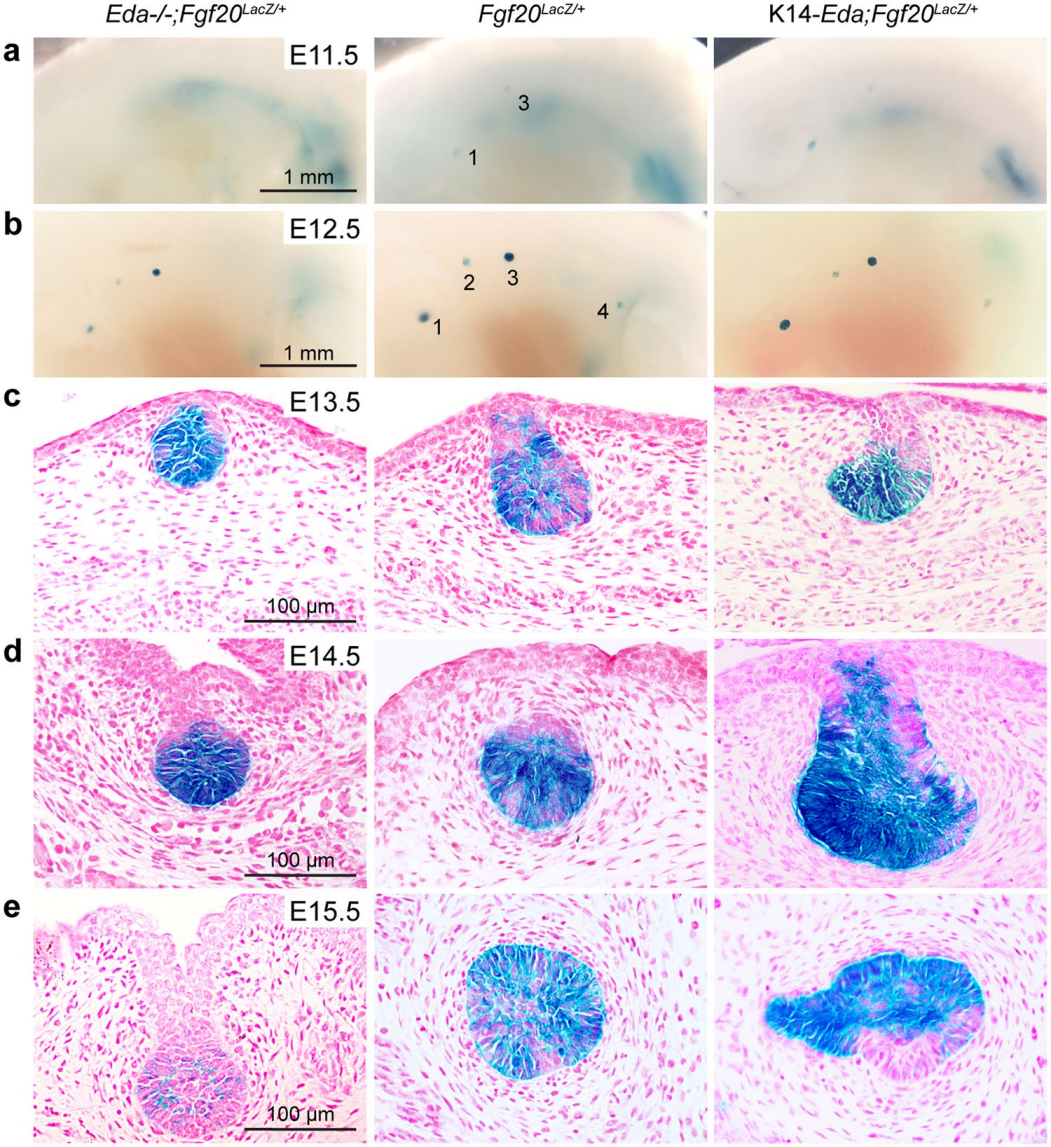
The influence of loss (*Eda* ^−/−^) and gain of Eda (K14-*Eda*) on the expression of *Fgf20-LacZ* in embryonic mammary glands. (**a**,**b**) Whole-mount X-Gal staining of *Eda* ^−/−^;*Fgf20* ^*LacZ*/+^; *Eda* ^+/+^;*Fgf20* ^*LacZ*/+^, and K14-*Eda*;*Fgf20* ^*LacZ*/+^ embryos at E11.5 and E12.5. (**c–e**) Histological sections of whole mount X-Gal stained mammary buds at E13.5 (mb2), E14.5 (mb2), and E15.5 (mb3).

The observation that Eda induced the expression of *Fgf20* in the embryonic mammary buds *ex vivo* prompted us to study the influence of Eda on *Fgf20* expression levels *in vivo* by analyzing the *Fgf20-LacZ* expression in *Eda* null and *Eda-*overexpressing (K14-*Eda*) embryos. In *Eda* ^−/−^ embryos there was a slight delay in the onset of *Fgf20-LacZ* expression at E11.5 followed by somewhat decreased signal at E12.5 compared to control or K14-*Eda* embryos (Fig. 2a,b). At E13.5–E14.5 expression in K14-*Eda* embryos appeared more intense (Fig. 2c,d), and at E15.5, *Fgf20-LacZ* expression levels correlated with the Eda status (Fig. 2e). Together, these data show that loss- and gain- of Eda influence *Fgf20-LacZ* expression, although modestly, yet clearly cues other than Eda have a more prominent impact on *Fgf20* expression during embryogenesis. The Wnt pathway is the most likely positive regulator: the murine *Fgf20* promoter is known to be highly responsive to β-catenin/Lef1 in promoter-reporter assays^39^.

### Absence of Fgf20 compromises mammary bud formation

**Figure 3:**
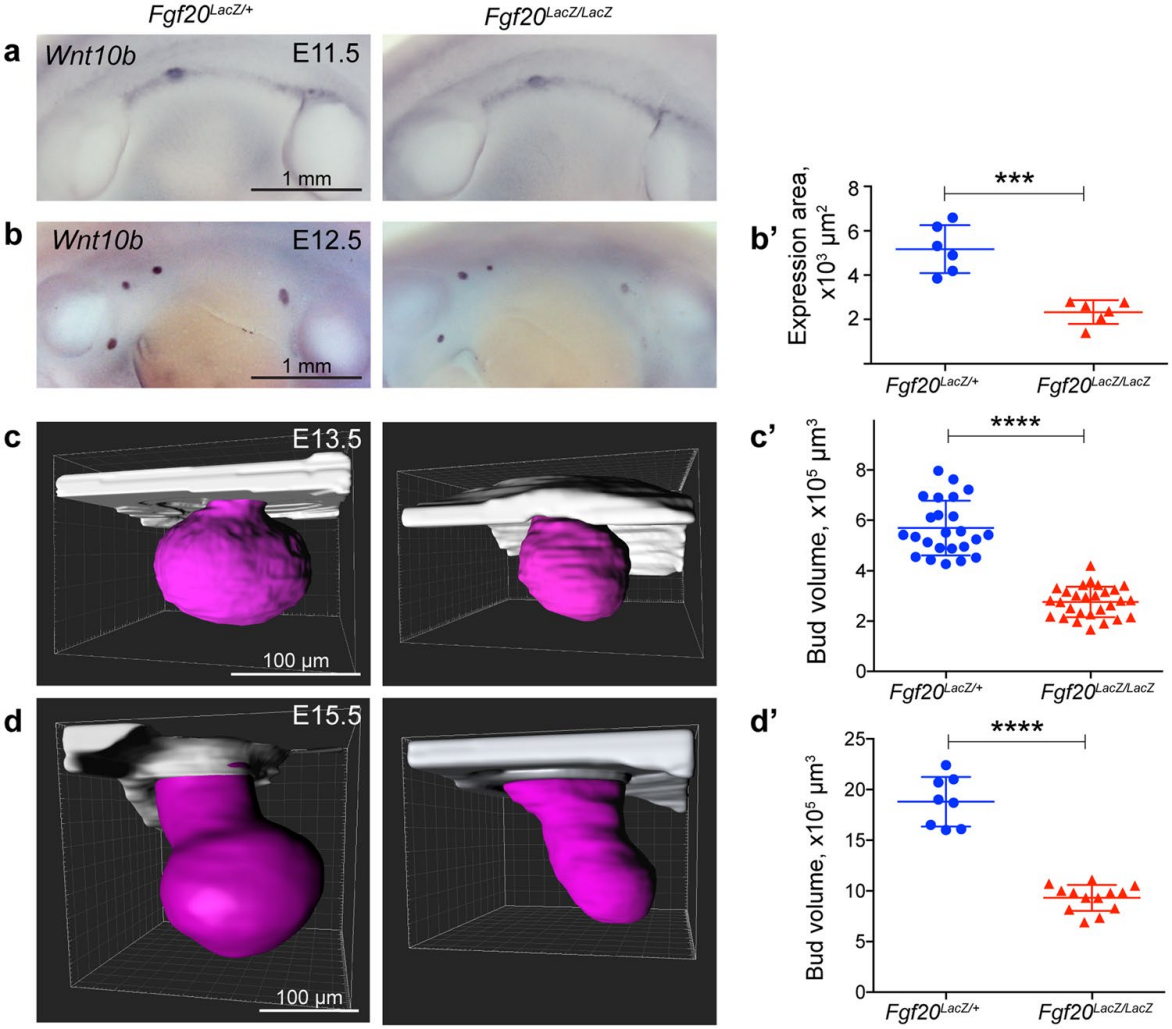
Fgf20 deficiency does not impede placode induction but compromises bud growth. (**a**) Expression of *Wnt10b* at somite stage 46–48 (*Fgf20* ^*LacZ*/+^, n = 7; *Fgf20* ^*LacZ/LacZ*^, n = 7) and (**b**) E12.5 (*Fgf20* ^*LacZ*/+^, n = 6; *Fgf20* ^*LacZ/LacZ*^, n = 6), and (**b’**) quantification of *Wnt10b* expression area (mammary bud 3) at E12.5. (**d,e**) 3D images and volume quantifications of EpCAM-stained mammary bud 3 at E13.5 (Fgf20^*LacZ*/+^, n = 24; *Fgf20* ^*LacZ/LacZ*^, n = 28), and E15.5 (Fgf20^*LacZ*/+^, n = 8; *Fgf20* ^*LacZ/LacZ*^, n = 13). The bud contours were outlined manually (purple) for volume quantification. ***p < 0.001; ****p < 0.0001.

**Figure 4:**
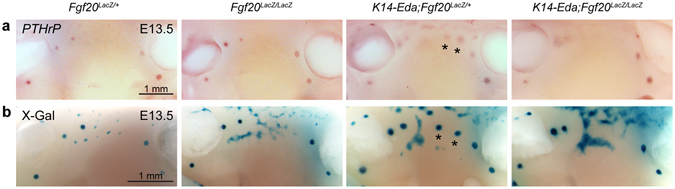
Fgf20 deficiency delays induction of supernumerary buds in K14-*Eda* mice. (**a**) Expression of *PTHrP* at E13.5 (*Fgf20* ^*LacZ*/+^, n = 4; *Fgf20* ^*LacZ/LacZ*^, n = 4; K14-*Eda;Fgf20* ^*LacZ*/+^, n = 7; K14-*Eda;Fgf20* ^*LacZ/LacZ*^, n = 5), and (**b**) X-gal staining of *Fgf20-LacZ* at E13.5 (*Fgf20* ^*LacZ/+*^, n = 4; *Fgf20* ^*LacZ/LacZ*^, n = 6; K14-*Eda;Fgf20* ^*LacZ/+*^, n = 11; K14-*Eda;Fgf20* ^*LacZ/LacZ*^, n = 8). Supernumerary placodes (stars) were detected between buds 3 and 4 in K14-*Eda*;Fgf20^*LacZ/+*^ embryos at E13.5, but not in K14-Eda;Fgf20^*LacZ/LacZ*^ embryos.

To elucidate the role of *Fgf20* in mammary gland development, we first analyzed the expression of placode markers *Wnt10b* and *PTHrP* by RNA *in situ* hybridization in the mammary buds of *Fgf20* ^*LacZ*/+^ and *Fgf20* ^*LacZ/LacZ*^ mice (Figs 3 and 4). At 46–48 somite stage (E11.5–E11.75) *Wnt10b* expression in the two genotypes was indistinguishable indicating that *Fgf20* deficiency does not impede induction of mammary gland development (Fig. 3a). At E12.5, however, *Wnt10b* expression domain appeared smaller in *Fgf20* ^*LacZ/LacZ*^ embryos, the difference being most pronounced in bud 3 (Fig. 3b), which is the first bud to form^11^. Quantification of the *Wnt10b* expression domain confirmed a significant difference between the two genotypes (p = 0.0007) (Fig. 3b’). At E13.5, the same was observed with the *PTHrP* probe, or when *Fgf20-LacZ* expression was assessed by X-gal staining (Fig. 4). For a more detailed morphological analysis, EpCAM-stained mammary buds 3 were visualized by whole mount confocal microscopy in 3D (Fig. 3c,d). Quantification revealed that *Fgf20* ^*LacZ/LacZ*^ buds were substantially smaller than control buds at E13.5 (p = 1.098E-13) and E15.5 (p = 2.234E-6). In attempt to gain insights into the molecular mechanisms underlying the *Fgf20* ^*LacZ/LacZ*^ bud phenotype, we analyzed expression of *Edar*, *Lef1*, and *Dkk4* at E12.5, and Lef1 protein at E13.5. No gross difference in *Edar*, or Lef1 expression was detected, but *Dkk4* expression was somewhat reduced in *Fgf20* ^*LacZ/LacZ*^ embryos (Supplementary Fig. S2), as previously shown in hair placodes (Huh *et al*.^39^).

The appearance of supernumerary mammary placodes between the endogenous buds 3 and 4 in K14-*Eda* mice has been shown at E12.5 by a number of Wnt pathway genes, and at E13.5 they are clearly visible by various mammary bud markers including *PTHrP* ^29, 33, 34^. At E13.5 *PTHrP* was not detected between bud 3 and 4 in K14-*Eda;Fgf20* ^*LacZ/LacZ*^ embryos in contrast to K14-*Eda;Fgf20* ^*LacZ*/+^ embryos (Fig. 4a). Furthermore, stereomicroscopic inspection, as well as X-gal staining and subsequent analysis of histological sections suggested the absence of supernumerary mammary buds in the K14-*Eda;Fgf20* ^*LacZ/LacZ*^ mice at E13.5 (Fig. 4b). However, based on similar analyses, supernumerary mammary buds were detectable in K14-*Eda;Fgf20* ^*LacZ/LacZ*^ embryos slightly later, at ~E14.0 (Supplementary Fig. S3). Accordingly, supernumerary nipples were observed on the milk line and the neck region of pre- and post-pubertal K14-*Eda;Fgf20* ^*LacZ/LacZ*^ females (Supplementary Fig. S3). As previously reported for K14-*Eda* males^34^, also K14-*Eda;Fgf20* ^*LacZ*/+^ and K14-*Eda*;*Fgf20* ^*LacZ/LacZ*^ males had supernumerary nipples, and at least in the neck region, a ductal tree was readily observed in compound mutants (Supplementary Fig. S3). In conclusion, in the absence of Fgf20, all mammary buds formed, yet a clear reduction in bud size and a slight delay in appearance of supernumerary mammary buds in K14-*Eda* embryos was evident.

### Absence of Fgf20 delays ductal growth in puberty

**Figure 5:**
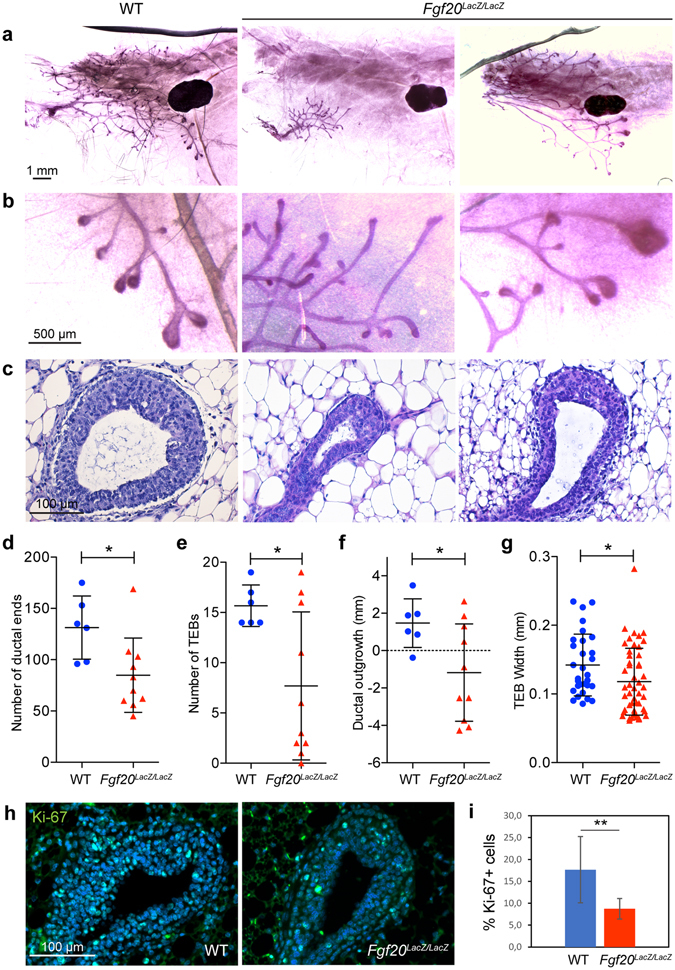
Fgf20 deficiency compromises TEB formation and ductal invasion. (**a–c**) Carmine alum stained ductal trees of the 4^th^ mammary gland (**a**,**b**) and histology of TEBs (**c**) of WT and *Fgf20* ^*LacZ/LacZ*^ mice at 5 weeks of age. (**d–g**) Quantification of the ductal ends (**d**), TEBs (**e**), ductal outgrowth (measured as the distance of furthest grown ductal end from the center of the lymph node) (**f**), and width of five biggest ductal ends in each gland (**f**) in WT (n = 6) and Fgf20^*LacZ/LacZ*^ (n = 10) mice. (**h**, **i**) Immunohistochemical staining and quantification of Ki-67 -positive cells in TEBs of WT (n = 4) and *Fgf20* ^*LacZ/LacZ*^ (n = 3) mice. Total number of TEBs analyzed was n = 15 (WT), n = 9 (*Fgf20* ^*LacZ/LacZ*^). Bars show mean ± SD. *p < 0.05; **p < 0.01.

Macroscopic analysis of pubertal and adult *Fgf20* ^*LacZ/LacZ*^ females revealed the presence of the normal number of nipples. To examine the impact of *Fgf20* deficiency on postnatal mammary morphogenesis, 4^th^ mammary glands of 5-week-old WT and *Fgf20* ^*LacZ/LacZ*^ were analyzed (Fig. 5a–c). The number of the ductal ends was reduced by 35% (p = 0.018) and TEBs by 51% (p = 0.008) in *Fgf20* ^*LacZ/LacZ*^ mice compared to WT controls (Fig. 5d,e). Also, the extent of ductal outgrowth (i.e. penetration to the fat pad) was significantly compromised (p = 0.037) (Fig. 5f). These data clearly show that absence of Fgf20 greatly retards ductal outgrowth during puberty. The ductal characteristics were, however, quite variable among the *Fgf20* ^*LacZ/LacZ*^ mice: often the ductal tree was very rudimentary and barely contained any TEBs while in some mice the ductal tree was only modestly affected (Fig. 5a–c). Quantification of the maximum width of the five largest TEBs/ductal tips in each specimen confirmed a significant difference between *Fgf20* ^*LacZ/LacZ*^ and WT mice (p = 0.029) (Fig. 5g). Ki-67 expression analysis in TEBs evidenced a decrease in the number of proliferating cells in *Fgf20* mutants (p = 0.0038) (Fig. 5h,i).

### No evidence for a systemic pubertal defect in Fgf20^LacZ/LacZ^ females

**Figure 6:**
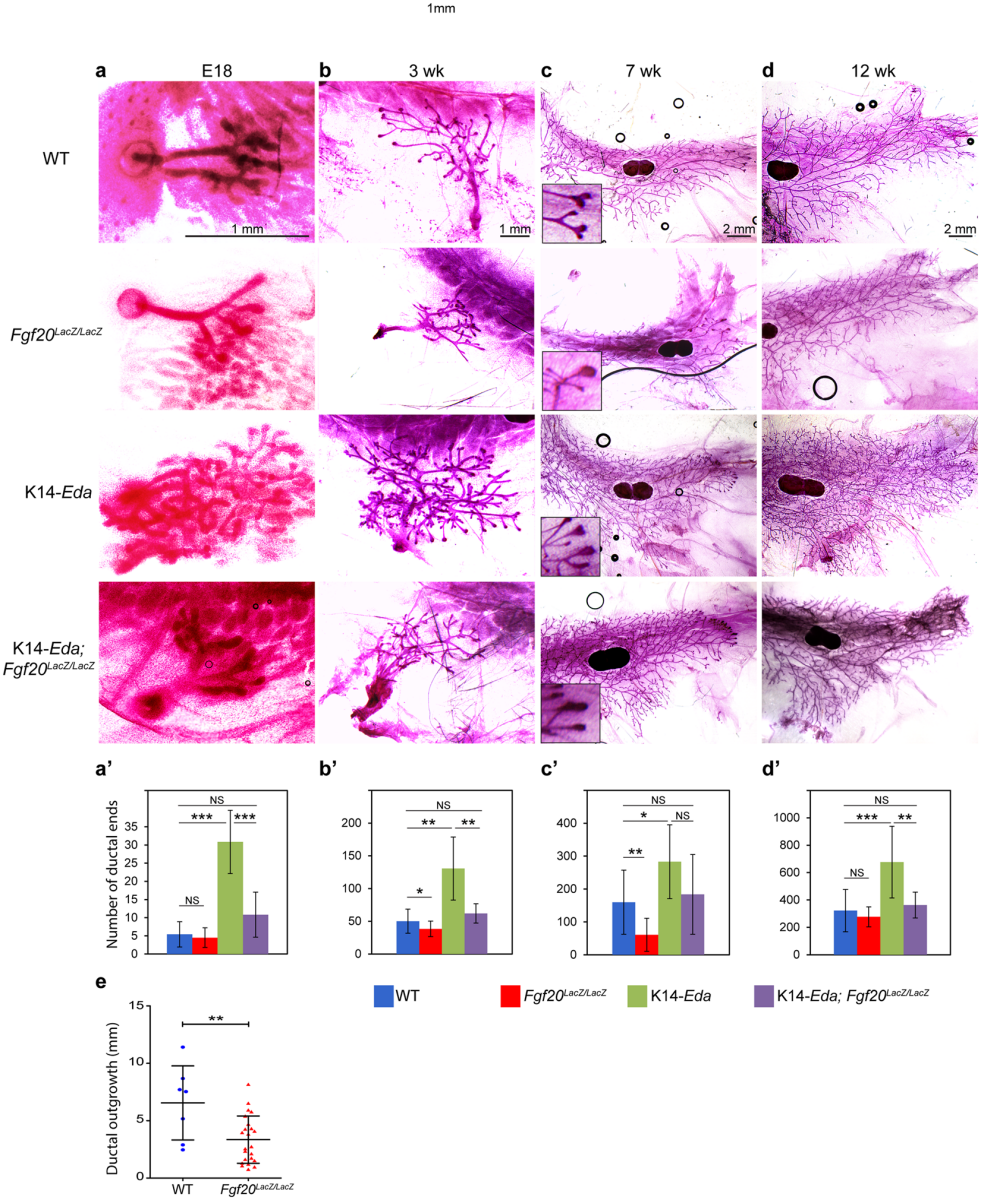
Loss of Fgf20 attenuates the K14-*Eda* hyperbranching phenotype. (**a–d**) Carmine alum stained 4^th^ mammary gland of WT, *Fgf20* ^*LacZ/LacZ*^, K14-*Eda*, and K14-*Eda;Fgf20* ^*LacZ/LacZ*^ mice at E18 (**a**), 3 weeks (**b**), 7 weeks (**c**), and 12 weeks of age (**d**). (**a’**–**d’**) Quantification of the total number of end ducts (**a’**,**b’**) or end ducts past the lymph node (**c’**,**d’**) in 4^th^ mammary gland. Number of glands analyzed were: WT (n_E18_ = 5, n_3wk_ = 18, n_7wk_ = 12, n_12wk_ = 15) *Fgf20* ^*LacZ/LacZ*^ (n_E18_ = 8, n_3wk_ = 16, n_7wk_ = 28, n_12wk_ = 5), K14-*Eda* (n_E18_ = 7, n_3wk_ = 8, n_7wk_ = 7, n_12wk_ = 13) and K14-*Eda;Fgf20* ^*LacZ/LacZ*^ (n_E18_ = 6, n_3wk_ = 8, n_7wk_ = 9, n_12wk_ = 10) (**e**) Ductal outgrowth (mm) measured from center of the lymph node in *Fgf20* ^+/+^ (n_glands_ = 7) and *Fgf20* ^*LacZ/LacZ*^ (n_glands_ = 23). Data are shown as mean ± SD. ***p < 0.001; **p < 0.01; *p < 0.05; NS, not significant.

We detected *Fgf20* expression only in the embryonic mammary glands (see above), yet F*gf20* ^*LacZ/LacZ*^ mammary glands displayed a remarkable postnatal phenotype (Figs 5 and 6). To assess whether the pubertal phenotype could be caused by a systemic defect due to the germline deletion of the *Fgf20* gene, we analyzed various parameters in the mutant animals. We found no difference in the onset of puberty, nor in the weight of the animals at the onset of, or during puberty (at 3, 5, or 7 weeks of age), or the weight of ovaries and uteri (Supplementary Fig. S4). Yet, 18% of 7-week-old *Fgf20* ^*LacZ/LacZ*^ females (n = 22) had completely closed vaginas, whereas a similar defect was not observed in WT mice (n = 9). These mice were not used for mammary gland analyses. The estrus cycles analyzed from vaginal smear cytology of WT and *Fgf20* ^*LacZ/LacZ*^ females were normal, and serum estradiol levels of the 7-week-old *Fgf20* ^*LacZ/LacZ*^ females in diestrus were similar to those of WT littermates (Supplementary Fig. S4). Finally, we performed mammary fat pad transplantations in which 1 mm^3^ pieces of adult *Fgf20* ^+/+^ mammary glands were transplanted into the cleared fat pad of 3-to-4-week old WT or *Fgf20* ^*LacZ/LacZ*^ females and allowed to grow for 5 weeks before analysis. WT epithelium grew equally well in the fat pad of both recipients (Supplementary Fig. S4). Collectively, these data indicate that there is no gross systemic defect in *Fgf20* ^*LacZ/LacZ*^ females, which could explain the pubertal mammary phenotype.

### Absence of Fgf20 normalizes the hyperbranching phenotype of K14-Eda mice

Our data showing that *Fgf20* expression levels are modulated by Eda (Fig. 1) and loss of *Fgf20* delays ductal growth at puberty (Fig. 5) prompted us to study the effects of *Fg20* deficiency on ductal branching at other developmental stages, as well as the crosstalk with the Eda pathway. At E18, the number of ductal ends in the mammary glands of *Fgf20* ^*LacZ/LacZ*^ embryos was similar to that of wildtype mice (p = 0.638) (Fig. 6a,a’). However, mammary glands of 3-week-old *Fgf20* ^*LacZ/LacZ*^ mice contained somewhat lower number of ductal tips than those of WT controls (p = 0.0321) (Fig. 6b,b’). At 7 weeks of age, the decrease in the ductal outgrowth and number of ductal ends in *Fgf20* ^*LacZ/LacZ*^ mice was prominent (p = 0.0039 and p = 0.0051, respectively) (Fig. 6c,c’,e), even more pronounced than at 5 weeks of age (Fig. 5d). However, at 12 weeks of age, the number of ductal ends was similar in both genotypes (p = 0.363) (Fig. 6d,d’).

Consistent with our previous results^34^, the number of ductal ends was significantly higher in K14-*Eda* mice compared to WT controls at E18 (p = 0.00009) and 3 weeks of age (p = 0.0019) (Fig. 6a,a’,b,b’). The hyperbranching phenotype was apparent also at 7 (p = 0.034) and 12 weeks of age (p = 0.0004) (Fig. 6c,c’,d,d’). Surprisingly, even though *Fgf20* null mammary glands did not display a growth phenotype at E18, the K14-*Eda* phenotype was greatly attenuated in *Fgf20* ^*LacZ/LacZ*^ background (p = 0.0005) (Fig. 6a’). Also at later stages, loss of *Fgf20* normalized the K14-*Eda* phenotype, although at 7 weeks of age, the difference did not reach statistical significance (p_3wk_ = 0.0046; p_7wk_ = 0.1521, p_12wk_ = 0.0011). These data identify Fgf20 as a critical mediator of Eda in mammary ductal growth and branching.

### At late puberty, the terminal end buds of Fgf20^LacZ/LacZ^ mice are larger and more proliferative

Since the growth delay of the *Fgf20* mutants was most pronounced at 7 weeks of age, we analyzed the ducts and TEBs of *Fgf20* ^*LacZ/LacZ*^ and WT glands in more detail at this stage. The architecture of the ducts appeared normal based on all criteria used: histology, hormone receptor expression, the distribution of basal (K14) and luminal (K8) keratins, and the expression of basal cell marker α-SMA (Supplementary Fig. S5). Accordingly, FACS analysis did not show significant differences in the percentage of luminal (CD29^lo^CD24+) or basal (CD29^hi^ CD24+) cells between WT and the *Fgf20* ^*LacZ/LacZ*^ mice at 7 week of age, nor at 3 weeks when the growth phenotype was first evident (Supplementary Fig. S5).

**Figure 7:**
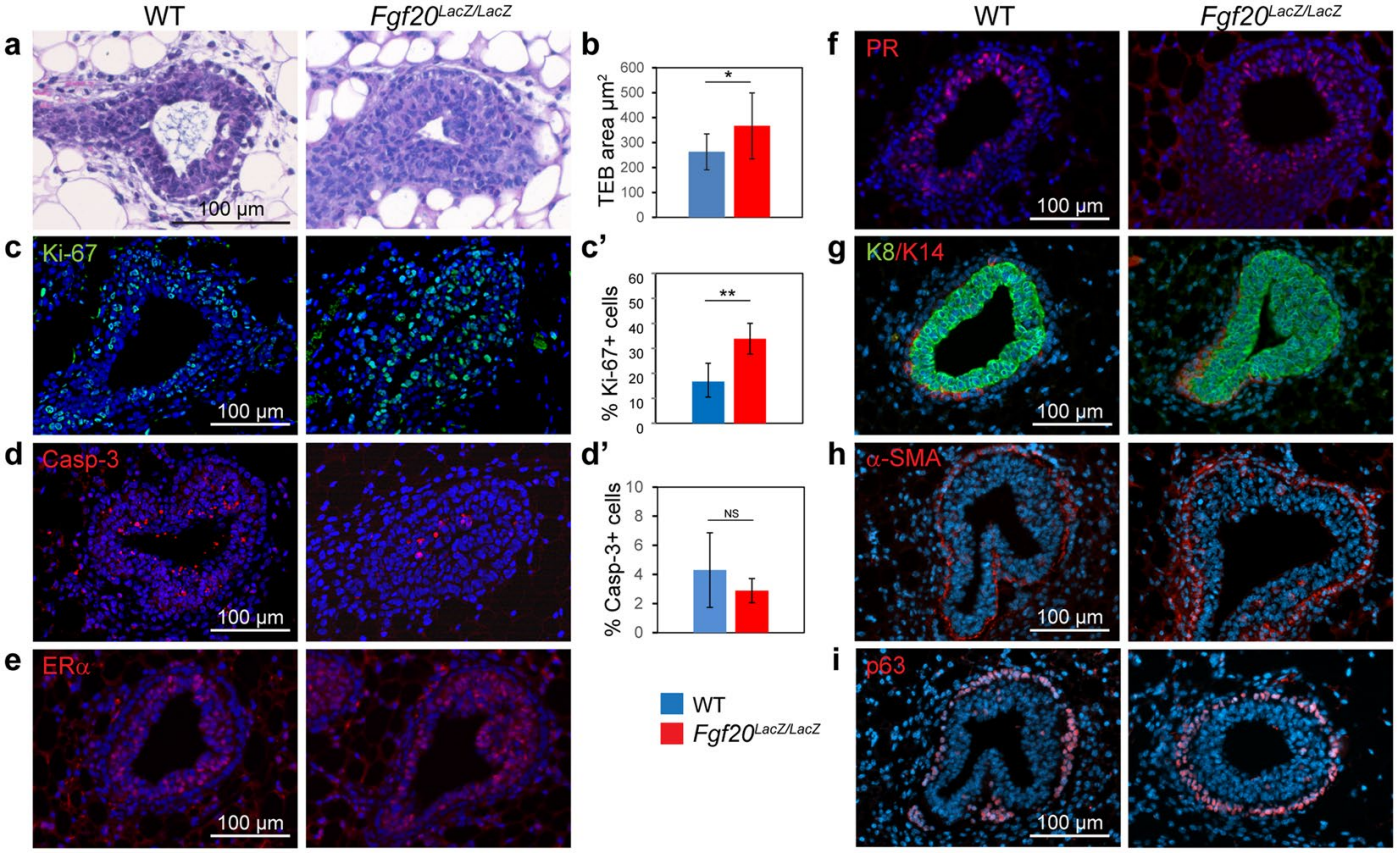
Analysis of terminal end buds of *Fgf20* ^*LacZ/LacZ*^ mice at 7 weeks of age. (**a**) Hematoxylin Eosin -stained sections of WT and *Fgf20* ^*LacZ/LacZ*^ TEBs. (**b**) Quantification of TEB area from Carmine alum stained mammary glands of WT (n = 9) and *Fgf20* ^*LacZ/LacZ*^ (n = 12) mice. (**c**,**c’**) Immunohistochemical staining and quantification of Ki-67 –positive cells in TEBs of WT (n = 4) and *Fgf20* ^*LacZ/LacZ*^ (n = 5) mice. Total number of TEBs analyzed was n = 26 (WT), n = 30 (*Fgf20* ^*LacZ/LacZ*^). (**d**,**d’**) Immunohistochemical staining and quantification of cleaved caspase-3 –positive cells in WT (n = 4) and *Fgf20* ^*LacZ/LacZ*^ mice (n = 4). Total number of TEBs analyzed was n = 34 (WT), n = 29 (*Fgf20* ^*LacZ/LacZ*^). (**e–i**) Immunohistochemical staining of ERα (**e**), PR (**f**), K8 and K14 (**g**), SMAα (**h**), and p63 (**i**) in the TEBs of WT and *Fgf20* ^*LacZ/LacZ*^ mice. Minimum of 4 mice per genotype were analyzed. Values represent mean ± SD. **p < 0.01; *p < 0.05; NS, not significant.

Analysis of TEBs, however, revealed that the epithelium appeared more cellular in *Fgf20* ^*LacZ/LacZ*^ mice compared to WT mice (Fig. 7a). TEB area, measured from the carmine alum whole mount images, was larger in *Fgf20* ^*LacZ/LacZ*^ mice at the same age (Fig. 7b). Quantification of Ki-67 and cleaved caspase-3 positive cells in TEBs revealed that the proportion of the proliferating cells was significantly higher in *Fgf20* ^*LacZ/LacZ*^ mice compared to WT controls (Fig. 7c,c’), but there was no difference in the proportion of apoptotic cells (Fig. 7d,d’). ERα and PR expression was indistinguishable between WT and *Fgf20* ^*LacZ/LacZ*^ TEBs (Fig. 7e,f). TEBs consist of a mass of luminal K8+ body cells surrounded by α-SMA+/p63+ cap cell layer. The expression patterns of body and cap cell markers were unchanged in 7-week old *Fgf20* ^*LacZ/LacZ*^ mice (Fig. 7g–i) indicating intact TEB architecture and cell identities.

## Discussion

In the current study, we have unveiled a role for Fgf20 in two stages of embryonic mammary gland development: budding and branching morphogenesis. Even though Fgf20 was dispensable for mammary placode induction, the buds were smaller in size. The molecular mechanism underlying the bud growth defect remain elusive. Furthermore, loss of Fgf20 delayed, but did not prevent, the formation of supernumerary mammary buds in K14-*Eda* embryos. Perinatally, *Fgf20* null mammary glands did not differ from the WT controls, yet the K14-*Eda* hyperbranching phenotype was greatly attenuated in *Fgf20* null background. The most plausible explanation for these seemingly contradictory findings is redundancy of Fgf20 with other Fgf ligands, the most prominent candidate being Fgf9, a member of the same Fgf subfamily. *Fgf9* is expressed in embryonic mammary glands^40^, shares similar biochemical properties with Fgf20 including receptor specificity^1, 2^, and redundancy between these two Fgfs has already been demonstrated in developing teeth^38^, kidney^42^, and cochlea^43^. Other Fgfs reported to be expressed in mammary bud epithelium are *Fgf4*, *Fgf8*, and *Fgf17* ^40^, which may further compensate for loss of *Fgf20*.

Fgf signaling typically mediates crosstalk across tissue compartments^1^, but whether the effects of Fgf20 on mammary gland epithelium are direct, mediated by the stroma, or both, is currently unknown. Fgf20 preferentially, but not exclusively, activates the mesenchymally expressed IIIc receptors isoforms^1, 2^. In the developing cochlea, epithelially expressed Fgf20 positively regulates epithelial progenitor proliferation via the mesenchyme, whereas intraepithelial Fgf20 signaling is essential for sensory cell differentiation^41, 43^. In hair follicles, Fgf20 is dispensable for placode formation, but is necessary for condensation of the underlying mesenchymal cells, which in turn is required for further follicular downgrowth^39^. The target genes regulated by Fgf20 have remained elusive in all organs studied so far.

We have previously shown that Eda regulates expression of *Fgf20* in embryonic hair follicles and teeth where Fgf20 functions as one of the major downstream effectors of the Eda pathway^38, 39^. Here, we identify Fgf20 as a mediator of Eda in the developing mammary glands: absence of Fgf20 delayed formation of supernumerary buds and normalized the hyperbranching mammary phenotype of K14-*Eda* mice, an effect maintained until adulthood. However, our data implicate the existence of other downstream targets of Eda besides Fgf20, since at E18 and at the onset of puberty, the ductal trees of *Eda*-null mice are more severely affected than those of *Fgf20* ^*LacZ/LacZ*^ mice^34^. Our earlier studies have identified several other Eda-induced factors that can enhance branching morphogenesis such as PTHrP, Egfr ligands, and Wnt pathway agonists^34^. Hence, the *Eda*-null and K14-*Eda* branching phenotypes are likely the combinatorial result of changes in the expression level of multiple Eda target genes.

Our data show that Fgf20 has a considerable impact on postnatal mammary morphogenesis since its absence led to defective TEB formation and delayed ductal invasion during puberty. However, the ductal growth defect was transient: the ductal trees caught up to the WT glands between 7 and 12 weeks of age. We propose that this also explains the counterintuitive finding of increased cell proliferation in *Fgf20* ^*LacZ/LacZ*^ TEBs at 7 weeks of age. In WT glands, the percentage of proliferative cells in the TEBs decreases between 3 weeks of age and late puberty (7 weeks)^26^, whereas *Fgf20* ^*LacZ/LacZ*^ mammary glands begin their growth burst at 7 weeks of age.

The embryonic phenotype and the subtle reduction in the number of branches in 3 weeks old *Fgf20* ^*LacZ/LacZ*^ mice implicates that the defect underlying the pubertal ductal phenotype may arise before puberty. We were unable to detect *Fgf20* expression during puberty, not even by qRT-PCR, a finding in line with a recent study assessing *Fgf20* expression in mammary glands of 3, 5, and 10 week old mice^27^. Thus, it is plausible that *Fgf20* deficiency during embryogenesis leads to qualitative changes in the mammary stem/progenitor cells that fully manifest only during puberty. Fittingly, a recent study implicated epithelial Fgfr1/2 signaling in proper mammary stem cell function during development^26^. However, we cannot exclude the possibility that *Fgf20* is expressed during puberty in a rare cell population that escaped our analysis. To answer the question whether Fgf20 has a role in pubertal development independent of its embryonic function must await for the generation of a conditional *Fgf20* mouse.

The mammary phenotype of *Fgf20* ^*LacZ/LacZ*^ mice resembles the phenotypes generated by K14-Cre-mediated deletion of Fgfr1^26^ and MMTV-Cre-mediated (mosaic) deletion of Fgfr2^24^, which both display compromised TEB formation, reduced number of branch points, and pubertal ductal outgrowth defect that normalizes in the adulthood. A complete failure in TEB maintenance is observed in mice inducibly overexpressing a transgene encoding a soluble form of Fgfr2b^25^. Interestingly, upon cessation of transgene expression 6 weeks after its induction, TEBs reform and branching is resumed. These data are suggestive of Fgfr signaling being essential for the functionality rather than survival of mammary stem/progenitor cells driving TEB formation and ductal invasion.

Pubertal ductal morphogenesis is a complex hormone regulated process, which involves cellular functions such as proliferation, apoptosis, migration, ECM degradation, and a tight interplay between epithelial and mesenchymal compartments^6, 15^. A great number of genetically manipulated mice, and experiments using slowly-releasing protein pellets *in vivo*, are known to cause a pubertal mammary phenotype^23, 44^. These studies show that loss of tissue integrity in TEBs readily leads to ductal outgrowth defects. However, this is unlikely the case in *Fgf20* ^*LacZ/LacZ*^ mice, as the expression pattern of body and cap cell markers was unaltered. Another important class of pubertal phenotypes is caused by loss- or gain-of-function of matrix remodeling enzymes such as matrix metalloproteinases (MMPs), which regulate ductal invasion and branching via their ability to sculpt the ECM^45, 46^, and Fgfr1/2 stimulation has been shown to induce the expression of *Mmp3* and *Mmp9* in several breast cancer and immortalized mammary epithelial cell lines^47–51^.

In conclusion, our results identify a hitherto unknown function for Fgf20 in both embryonic and postnatal mammary gland morphogenesis. Our data suggest that compromised Fgf20 signaling during embryogenesis results in qualitative changes in TEBs that are thought to harbor the majority of stem cells driving branching morphogenesis during puberty^52, 53^. To our knowledge, in addition to Fgf10^11^, Fgf20 is the only Fgf family member with a proven *in vivo* function in mammary gland development. Furthermore, we discovered Fgf20 as an important mediator of Eda in mammary gland budding and branching morphogenesis. Future studies should shed light on the molecular mechanisms downstream of Fgf20 in mammary gland morphogenesis.

## Materials and Methods

### Mice

The generation and genotyping of Fgf20^*LacZ/LacZ*^, K14-*Eda*, and *Eda* ^−/−^ (*Tabby*; Jackson Laboratories, stock no 000314) mouse strains have been described previously^41, 54^. *Fgf20* ^*LacZ/LacZ*^ and K14-*Eda* mice were maintained in the C57Bl/6 background (K14-*Eda* > 10 generations and *Fgf20* ^*LacZ/LacZ*^ > 5 generations) and *Eda* ^−/−^ mice in the B6CBA background. Embryonic ages were defined based on the appearance of vaginal plug and external criteria including limb morphogenesis^55^. The sex of embryos older than E14 was defined by PCR with *Sry*-specific primers or anatomy, and only female mice were used for analysis unless otherwise stated. All mouse studies were approved and carried out in accordance with the guidelines of the Finnish national animal experimentation board.

### Embryonic mammary bud cultures and quantitative RT-PCR

The hanging drop culture method used for the embryonic mammary bud cultures has been described in detail previously^33, 34^. Pooled (15–20 buds per pool) E13.5 *Eda* ^−/−^ mammary buds were treated with 250 ng/ml of Fc-Eda^56^ or control medium, RNA was extracted, cDNA synthesized, and qRT-PCR performed with the Light Xycler480 machine (Roche, Indianapolis, IA) as described^33, 34^ using the following primers:

*Fgf4F* 5′-CGAGGGACAGTCTTCTGGAG-3′, *Fgf4*R 5′-GTACGCGTAGGCTTCGTAGG-3′, *Fgf9*F 5′-GGGGAGCTGTATGGATCAGA-3′, *Fgf9*R 5′-CTTTGTCAGGGTCCACTGGT-3′ *Fgf17F* 5′-GACAGATACATTCGGCAGCA-3′, *Fgf17*R 5′-CTGGAAGGCCGTGTAGTTGT-3′, *Fgf20F 5′-*GTGCCAGGTCCAAAAGACAT-3′, *Fgf20*R 5′-GGAGAATGATCTTGCTTTGCTT-3′. Dilution series of PCR products was used for quantifying the transcript numbers of genes of interest with the help of Lightcycler480 software. *Ranbp1* (F 5′-ACGCTGGAGGAAGATGAAGA-3′, and R 5′-TCATAAGAAGGCGGATGGTC-3′) or *GAPDH* (F 5′-CTCGTCCCGTAGACAAAATGG-3′ and R 5′-AGATGGTGATGGGCTTCCC-3′) were used as a reference genes.

### X-Gal and Carmine alum staining

X-Gal staining for whole embryos (E10.5–E15.5) or abdominal skins of embryos (E16.5–E18) was performed according to a published protocol^32^ with an overnight incubation in the 1 mg/ml X-Gal substrate. For postnatal mammary glands, a modified X-Gal-staining method was used^57^. The X-Gal stained samples were post-fixed with 4% PFA, dehydrated, embedded in paraffin and counterstained with nuclear fast red after sectioning. For quantification of mammary ductal ends, the 4^th^ mammary glands and surrounding fat pads of E18, 3-, 5-, 7- and 12-week old mice were prepared, spread on slides and subjected to Carmine alum staining as previously described^34^. Quantification of ductal ends, TEBs, and the areas and maximum widths of TEBs was done manually from images with the help of Fiji ImageJ software. Ductal outgrowth was measured as the distance of the furthest grown ductal end from the center of the lymph node.

### *In situ* hybridization

For whole mount *in situ* hybridization with digoxigenin-labeled RNA probes, E11.5–E13.75 embryos were fixed in 4% PFA overnight at 4 °C and dehydrated with rising methanol series. *In situ* hybridization was performed with inSituPro robot (Intavis AG) as previously published^29, 38^ or manually using a similar protocol. The digoxigenin-labeled RNA probes for *Wnt10b*, *Edar*, *Dkk4*, *Lef1, PTHrP* and *Fgf20* have been described earlier^34^; *Fgf20* probe corresponded to the open reading frame. BM Purple AP substrate Precipitating Solution (Boehringer Mannheim) was used for detection of digoxigenin-labeled RNA probes. Radioactive *in situ* hybridization was performed on paraffin sections using ^35^S-UTP labeled (Amersham) probe specific to *Fgf20* as described^38^.

### Immunohistochemical stainings

For immunohistochemical and hematoxylin-eosin stainings, the 4^th^ mammary glands of WT and *Fgf20* ^*LacZ/LacZ*^ mice were dissected, spread on microscope slides, and fixed with 4% PFA overnight at 4 °C. Alternatively, 13.5 trunks were dissected. The samples were dehydrated, embedded in paraffin, and 5 µm sections were cut. Slides were deparaffinized by standard methods. In immunohistochemical stainings antigen retrieval was performed by heating the slides in microwave oven in TE buffer, pH 9.0 (keratin-8 (K8), keratin-14 (K14), progesterone receptor (PR) and estrogen receptor α (ERα) stainings), or in 10 mM sodium citrate buffer pH 6.0 (β-Galactosidase, cleaved Caspase-3, Ki-67, α-smooth muscle actin (α-SMA), Lef1, and p63 stainings). Primary and secondary antibodies used are listed in Supplementary information. Samples were imaged with a Zeiss Axio Imager M2 microscope equipped with an AxioCam HRc camera (Zeiss) and processed in Photoshop.

### Mammary bud area and volume quantification

*Wnt10b* expression area was quantified manually from images with the help of Fiji ImageJ software. For whole-mount immunofluorescence staining E13.5 and E15.5 mouse embryos were fixed in 4% PFA at 4 °C overnight. After washing the samples with PBS for 3–4 hours, they were permeabilized with 0.3% TritonX-100 in PBS for 1–2 hours at room temperature, blocked (5% normal donkey serum, 0.5% BSA, and 0.3% TritonX-100 in PBS) for 1 h, and incubated at 4 °C with rat anti-mouse CD326 (EpCAM, BD Pharmingen, 552370, 1:1,000) and 10 µg/ml Hoechst 33258 (Molecular Probes/Invitrogen) in blocking buffer for 2 days. EpCAM was detected with an Alexa Fluor 647 –conjugated secondary antibody (Molecular Probes/Invitrogen). The ventral skin around mammary gland 2 and 3 was dissected and mounted with Vectashield (Vector Laboratories) and visualized using a Zeiss LSM700 laser scanning confocal microscope. For mammary placode and bud volume quantification, the area of mammary primordium was outlined manually based on EpCAM expression and bud morphology. Surface rendering and volume quantification were performed with Imaris 8.3 software (Bitplane).

### Mammary cell preparation, cell labelling, and flow cytometry

Single cell suspension of mammary gland was prepared according to the protocol modified from Shackleton *et al*.^58^. Briefly, the 4^th^ mammary glands were cut into small pieces after removal of the lymph node. The tissues were digested in a mixture of 5 ml collagenase I buffer (10% FBS, 100 mg/ml streptomycin, 10 U/ml penicillin, 300 U/ml collagenase I (ThermoFisher) and 100 U/ml hyaluronidase (Sigma) in DMEM/F12 (1:1) medium for 1–2 hours at 37 °C with moderate shaking. The cell suspension was washed in PBS and digested further in 0.25% trypsin-EDTA for 5–10 minutes. The red blood cells were removed by incubation in red blood cell lysing buffer (Biolegend) on ice for 5 minutes. The single cell suspension was passed through 40 µm cell strainer (BD Bioscience) before stained with the mixture of antibodies on ice for 30 minutes. After washing in PBS, the dead cells were labeled with Fixable Viability Dye eFluor 780 (eBioscience) for 30 minutes on ice. Flow cytometry was carried out by BD LSR II, and data analysis was done by Flowjo. The following antibodies were used: CD45-PE (Biolegend, 103106, 1:200), CD31-PE (Biolegend, 102507, 1:200), TER119-PE (Biolegend, 116207, 1:200) CD24-PeCy7 (Biolegend, 101822, 1:200) and CD29-APC (eBioscience, 17–0291–82, 1:200).

### Monitoring the onset of puberty, estrous phase, and measurement of estradiol

Onset of puberty was assessed by monitoring the vaginal opening (VO) by visual examination of vulva^59^ every morning 5 days/week (Mon–Fri) starting at the age of 18 days until the appearance of VO. In case of VO occuring during the weekend, the earliest, latest, and average times of VO were defined and separate comparisons of *Fgf20* ^*LacZ/LacZ*^ and WT mice were done using average VO time as well as extreme VO times (eg. VO_WTlatest_ vs. VO_*Fgf20LacZ/LacZ* earliest_ and vice versa).

Estrus phase was defined by examining the vaginal cytology collected by vaginal lavage with PBS using a small pipet and stained by crystal blue as previously described^60^. For monitoring the regularity of estrus cycles, 7-week and 12-week-old Fgf20^*LacZ/LacZ*^ and WT females were examined 5 days/week in the mornings for at least two weeks.

Estradiol levels were measured from serum of 7-week-old mice in diestrus by highly sensitive gas chromatography-tandem mass spectrometry^61^. In case of obtaining zero value from the measurement (n = 3 in both WT and Fgf20^*LacZ/LacZ*^), value equal to ½ LLOD (lower limit of detection) of estradiol (0.15 pg/ml) was used for the sample^61^. Mice were sacrificed, blood samples were immediately taken by heart puncture and kept overnight at 4 °C. Mammary glands were used for FACS analysis and immunohistochemistry and uterus and ovaries were carefully dissected and weighted. Serum was dissociated the following day by centrifugation in at 3000 rpm at 4 °C. Minimum of 250 µl of serum was required for mass spectrometry analysis.

### Mammary fat pad transplantations

For mammary fat pad transplantations, 3–4 week old WT (n = 6) and *Fgf20* ^*LacZ/LacZ*^ (n = 5) recipient females were anesthetized and the fat pad of left 4^th^ mammary gland was cleared until the lymph node as described^62^. ~1 mm^3^ pieces of adult (12–13-week-old) WT donor (n = 4) mammary glands were transplanted into cleared fat pads. Five weeks later transplanted mammary glands were collected, stained by Carmine alum, and ductal ends quantified.

### Statistical analysis

P-values were calculated with unpaired t-test assuming unequal variances unless otherwise stated.

### Data availability

The datasets generated during the current study are available from the corresponding author on reasonable request.

## Electronic supplementary material

Elo et al_Supplementary Info

## References

[1] Ornitz DM, Itoh N (2015). The Fibroblast Growth Factor signaling pathway. Wiley Interdiscip. Rev. Dev. Biol..

[2] Zhang X (2006). Receptor specificity of the fibroblast growth factor family. The complete mammalian FGF family. J. Biol. Chem..

[3] Macias H, Hinck L (2012). Mammary gland development. WIREs Dev. Biol..

[4] Propper AY, Howard BA, Veltmaat JM (2013). Prenatal morphogenesis of mammary glands in mouse and rabbit. J. Mammary Gland Biol. Neoplasia.

[5] Sternlicht MD, Kouros-Mehr H, Lu P, Werb Z (2006). Hormonal and local control of mammary branching morphogenesis. Differentiation.

[6] Howard BA, Lu P (2014). Stromal regulation of embryonic and postnatal mammary epithelial development and differentiation. Semin. Cell. Dev. Biol..

[7] Cowin P, Wysolmerski J (2010). Molecular mechanisms guiding embryonic mammary gland development. Cold Spring Harb. Perspect. Biol..

[8] Biggs LC, Mikkola ML (2014). Early inductive events in ectodermal appendage morphogenesis. Semin. Cell. Dev. Biol..

[9] van Genderen C (1994). Development of several organs that require inductive epithelial-mesenchymal interactions is impaired in LEF-1-deficient mice. Genes Dev..

[10] Chu EY (2004). Canonical WNT signaling promotes mammary placode development and is essential for initiation of mammary gland morphogenesis. Development.

[11] Mailleux AA (2002). Role of FGF10/FGFR2b signaling during mammary gland development in the mouse embryo. Development.

[12] Hens JR (2007). BMP4 and PTHrP interact to stimulate ductal outgrowth during embryonic mammary development and to inhibit hair follicle induction. Development.

[13] Lindvall C (2009). The Wnt co-receptor Lrp6 is required for normal mouse mammary gland development. PLoS One.

[14] Sternlicht MD (2005). Mammary ductal morphogenesis requires paracrine activation of stromal EGFR via ADAM17-dependent shedding of epithelial amphiregulin. Development.

[15] Gjorevski N, Nelson CM (2011). Integrated morphodynamic signalling of the mammary gland. Nat. Rev. Mol. Cell. Biol..

[16] Sebastian J (1998). Activation and function of the epidermal growth factor receptor and erbB-2 during mammary gland morphogenesis. Cell Growth Differ..

[17] Wiesen JF, Young P, Werb Z, Cunha GR (1999). Signaling through the stromal epidermal growth factor receptor is necessary for mammary ductal development. Development.

[18] Ciarloni L, Mallepell S, Brisken C (2007). Amphiregulin is an essential mediator of estrogen receptor alpha function in mammary gland development. Proc. Natl. Acad. Sci. USA.

[19] Luetteke NC (1999). Targeted inactivation of the EGF and amphiregulin genes reveals distinct roles for EGF receptor ligands in mouse mammary gland development. Development.

[20] Mallepell S, Krust A, Chambon P, Brisken C (2006). Paracrine signaling through the epithelial estrogen receptor alpha is required for proliferation and morphogenesis in the mammary gland. Proc. Natl. Acad. Sci. USA.

[21] Ruan W, Kleinberg DL (1999). Insulin-like growth factor I is essential for terminal end bud formation and ductal morphogenesis during mammary development. Endocrinology.

[22] Gallego MI (2001). Prolactin, growth hormone, and epidermal growth factor activate Stat5 in different compartments of mammary tissue and exert different and overlapping developmental effects. Dev. Biol..

[23] McNally S, Martin F (2011). Molecular regulators of pubertal mammary gland development. Ann. Med..

[24] Lu P, Ewald AJ, Martin GR, Werb Z (2008). Genetic mosaic analysis reveals FGF receptor 2 function in terminal end buds during mammary gland branching morphogenesis. Dev. Biol..

[25] Parsa S (2008). Terminal end bud maintenance in mammary gland is dependent upon FGFR2b signaling. Dev. Biol..

[26] Pond AC (2013). Fibroblast growth factor receptor signaling is essential for normal mammary gland development and stem cell function. Stem Cells.

[27] Zhang X (2014). FGF ligands of the postnatal mammary stroma regulate distinct aspects of epithelial morphogenesis. Development.

[28] Mikkola ML (2009). Molecular aspects of hypohidrotic ectodermal dysplasia. Am. J. Med. Genet..

[29] Mustonen T (2004). Ectodysplasin A1 promotes placodal cell fate during early morphogenesis of ectodermal appendages. Development.

[30] Chang SH, Jobling S, Brennan K, Headon DJ (2009). Enhanced Edar signalling has pleiotropic effects on craniofacial and cutaneous glands. PLoS One.

[31] Kamberov YG (2013). Modeling recent human evolution in mice by expression of a selected EDAR variant. Cell.

[32] Pispa J, Pummila M, Barker PA, Thesleff I, Mikkola ML (2008). Edar and troy signalling pathways act redundantly to regulate initiation of hair follicle development. Hum. Mol. Genet.

[33] Voutilainen M (2015). Ectodysplasin/NF-κB promotes mammary cell fate via Wnt/β-catenin pathway. PLoS Genet..

[34] Voutilainen M (2012). Ectodysplasin regulates hormone-independent mammary ductal morphogenesis via NF-κB. Proc. Natl. Acad. Sci. USA.

[35] Clarke A, Phillips DI, Brown R, Harper PS (1987). Clinical aspects of X-linked hypohidrotic ectodermal dysplasia. Arch. Dis. Child..

[36] Lindfors PH, Voutilainen M, Mikkola ML (2013). Ectodysplasin/NF-κB signaling in embryonic mammary gland development. J. Mammary Gland Biol. Neoplasia.

[37] Lefebvre S, Fliniaux I, Schneider P, Mikkola ML (2012). Identification of ectodysplasin target genes reveals the involvement of chemokines in hair development. J. Invest. Dermatol..

[38] Häärä O (2012). Ectodysplasin regulates activator-inhibitor balance in murine tooth development through Fgf20 signaling. Development.

[39] Huh SH (2013). Fgf20 governs formation of primary and secondary dermal condensations in developing hair follicles. Genes Dev..

[40] Eblaghie MC (2004). Interactions between FGF and Wnt signals and Tbx3 gene expression in mammary gland initiation in mouse embryos. J. Anat..

[41] Huh SH, Jones J, Warchol ME, Ornitz DM (2012). Differentiation of the lateral compartment of the cochlea requires a temporally restricted FGF20 signal. PLoS Biol..

[42] Barak H (2012). FGF9 and FGF20 maintain the stemness of nephron progenitors in mice and man. Dev. Cell.

[43] Huh SH, Warchol ME, Ornitz DM (2015). Cochlear progenitor number is controlled through mesenchymal FGF receptor signaling. Elife.

[44] Howlin J, McBryan J, Martin F (2006). Pubertal mammary gland development: insights from mouse models. J. Mammary Gland Biol. Neoplasia.

[45] Fata JE, Ho AT, Leco KJ, Moorehead RA, Khokha R (2000). Cellular turnover and extracellular matrix remodeling in female reproductive tissues: functions of metalloproteinases and their inhibitors. Cell. Mol. Life Sci..

[46] Wiseman BS (2003). Site-specific inductive and inhibitory activities of MMP-2 and MMP-3 orchestrate mammary gland branching morphogenesis. J. Cell Biol..

[47] Hajitou A (1998). FGF-3 and FGF-4 elicit distinct oncogenic properties in mouse mammary myoepithelial cells. Oncogene.

[48] Ruohola JK (2001). Enhanced invasion and tumor growth of fibroblast growth factor 8b-overexpressing MCF-7 human breast cancer cells. Cancer Res..

[49] Liu JF, Crépin M, Liu JM, Barritault D, Ledoux D (2002). FGF-2 and TPA induce matrix metalloproteinase-9 secretion in MCF-7 cells through PKC activation of the Ras/ERK pathway. Biochem. Biophys. Res. Commun..

[50] Suyama K, Shapiro I, Guttman M, Hazan RB (2002). A signaling pathway leading to metastasis is controlled by N-cadherin and the FGF receptor. Cancer Cell.

[51] Xian W, Schwertfeger KL, Vargo-Gogola T, Rosen JM (2005). Pleiotropic effects of FGFR1 on cell proliferation, survival, and migration in a 3D mammary epithelial cell model. J. Cell Biol..

[52] Williams JM, Daniel CW (1983). Mammary ductal elongation: differentiation of myoepithelium and basal lamina during branching morphogenesis. Dev. Biol..

[53] Scheele CL (2017). Identity and dynamics of mammary stem cells during branching morphogenesis. Nature.

[54] Mustonen T (2003). Stimulation of ectodermal organ development by Ectodysplasin-A1. Dev. Biol..

[55] Martin P (1990). Tissue patterning in the developing mouse limb. Int. J. Dev. Biol..

[56] Gaide O, Schneider P (2003). Permanent correction of an inherited ectodermal dysplasia with recombinant EDA. Nat. Med.

[57] Visbal AP (2011). Altered differentiation and paracrine stimulation of mammary epithelial cell proliferation by conditionally activated Smoothened. Dev. Biol..

[58] Shackleton M (2006). Generation of a functional mammary gland from a single stem cell. Nature.

[59] Caligioni, C.S. Assessing reproductive status/stages in mice. Curr. Protoc. Neurosci. Appendix–4I, doi:10.1002/0471142301.nsa04is48 (2009). 10.1002/0471142301.nsa04is48 PMC2755182 19575469

[60] McLean AC, Valenzuela N, Fai S, Bennett SA (2012). Performing Vaginal Lavage, Crystal Violet Staining, and Vaginal Cytological Evaluation for Mouse Estrous Cycle Staging Identification. J. Vis. Exp.

[61] Nilsson ME (2015). Measurement of a comprehensive sex steroid profile in rodent serum by high-sensitive gas chromatography-tandem mass spectrometry. Endocrinology.

[62] Brill B, Boecher N, Groner B, Shemanko CS (2008). A sparing procedure to clear the mouse mammary fat pad of epithelial components for transplantation analysis. Lab. Anim..

