# Supplementary material for: Ectodysplasin target gene Fgf20 regulates mammary bud growth and ductal invasion and branching during puberty
Source: Sci Rep. 2017 Jul 11;7:5049. doi: 10.1038/s41598-017-04637-1 (PMC5505952; doi:10.1038/s41598-017-04637-1)
Supplement: Supplementary file 1 — Elo et al_Supplementary Info [file 41598_2017_4637_MOESM1_ESM.pdf]

Supplementary information

Ectodysplasin target gene Fgf20 regulates mammary bud growth and ductal invasion and branching during puberty

Teresa Elo, Päivi H. Lindfors, Qiang Lan, Maria Voutilainen, Ewelina Trela, Claes Ohlsson, Sung-Ho Huh, David M. Ornitz, Matti Poutanen, Beatrice A. Howard, Marja L. Mikkola

Supplementary Materials and Methods

Antibodies used in immunohistochemical stainings

Primary antibodies and dilutions used were: Rabbit anti-β-Galactosidase antibody, (MP Biomedicals, 55976), 1:1500; rabbit anti-Ki-67 (Abcam, ab1667) 1:100; rabbit anti-Caspase-3 **(**Cell Signaling Technology, 9661S), 1:6000; rat anti-K8 (DSHB, Troma1), 1:100; rabbit-anti-K14 (Neomarkers, RB-9020-P), 1:500; mouse anti-ERα (Dako, M7047), 1:50; mouse anti-PR (Thermo Scientific, MS-197-P1), 1:200; mouse anti-αSMA (Abcam ab7817), 1:150; rabbit anti-Lef1 (Cell Signaling Technology, 2230S), 1:500; and mouse anti-p63 (NeoMarkers, Clone 4A4, MS1081-P), 1:250,. Primary antibody incubations were carried out overnight at 4°C, except for anti-β-Galactosidase, which was incubated overnight at RT. Anti-K14 and K8 stainings were performed as double stainings, other were single stainings. The following secondary antibodies (Jackson ImmunoResearch) in the dilution of 1:500 were used: AlexaFluor 568 -conjugated goat-anti-mouse (PR and ERα, αSMA, p63), Alexa Fluor 568 –conjugated donkey-anti-rabbit (β-Galactosidase, Caspase-3), Alexa Fluor 695 -conjugated donkey anti-rabbit (K14), Alexa Fluor 544 -conjugated donkey anti-rat (K8) Alexa Fluor 488–conjugated donkey-anti-rabbit (Ki-67). Nuclear staining was achieved by using DAPI or Hoechst. In case of anti-β-Galactosidase staining, HRP-conjugated secondary Goat anti-rabbit antibody (Jackson Immuno Research, 111-035-045, 1:500) and detection with 3,3’ diaminobenzidine was also used.

Supplementary Figures

Figure S1.

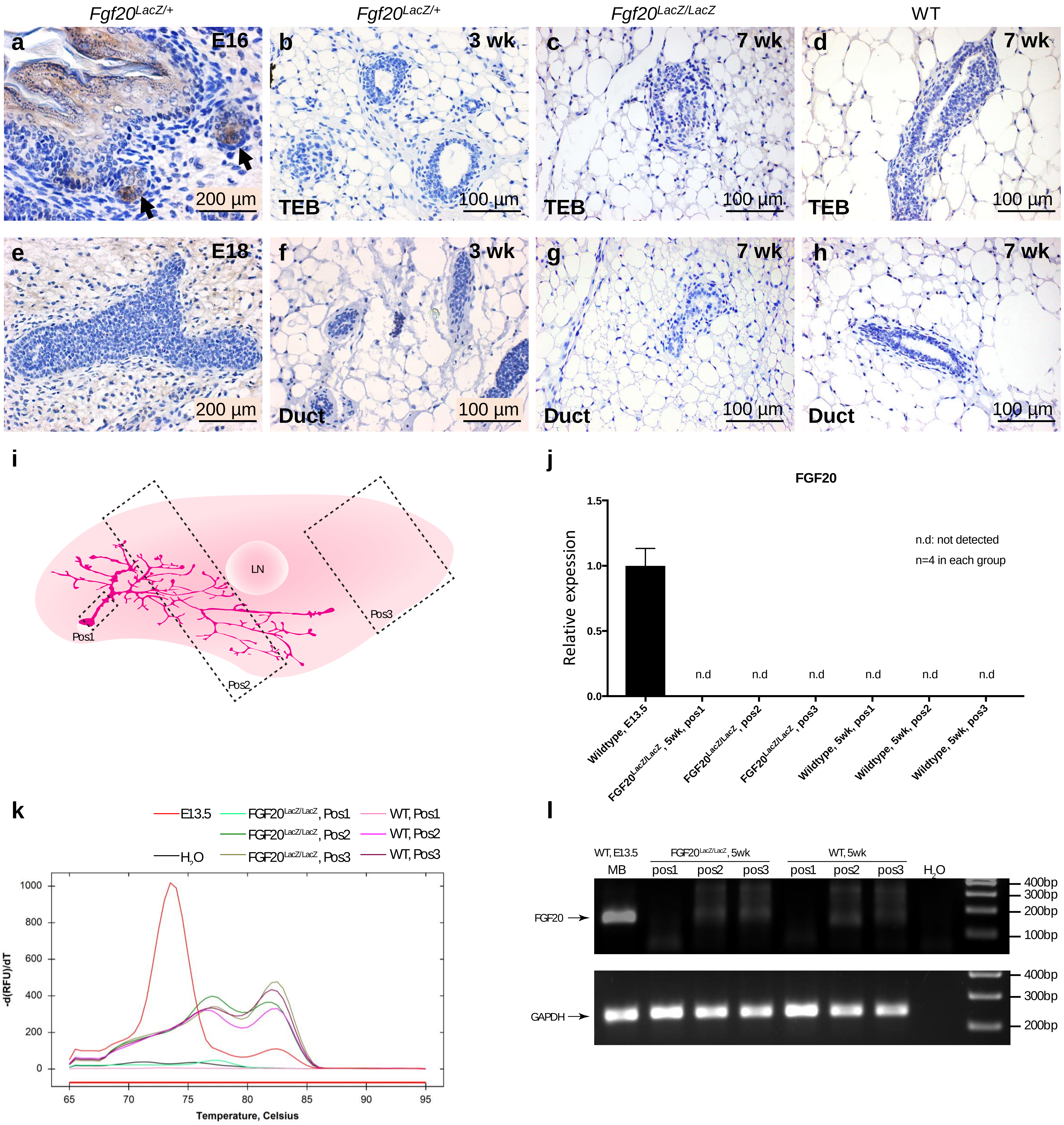

Supplementary Figure S1. Fgf20 expression analysis in postnatal mammary glands. Positive X-gal staining was detected in embryonic (E16) hair follicle (arrows) (**a**) but not in the late embryonic (E18) mammary glands of the *Fgf20*$^{LacZ/+}$ mice (**e**). No signal was detected in the mammary gland of 3-week-old *Fgf20*$^{LacZ/+}$ (**b**,**f**), or 7-week-old *Fgf20*$^{LacZ/LacZ}$ mice (**c**,**g**). Mammary glands of 7-week-old WT mice (**d**,**h**) served as a negative control. (**i-l)** qRT-PCR analysis of *Fgf20* in 5-wk old mammary gland 4. **i)** Schematic illustration of the location of samples analyzed (Pos1-3). **j)** Relative expression of *Fgf20* in mammary glands of 5-week-old WT and *Fgf20*$^{LacZ/LacZ}$ mice quantified by qRT-PCR (n=4 in each sample). Mammary buds of WT E13.5 were used as positive controls. **k)** Representative example of melting curves of qPCR products presented in (Fig. S1**j**). **l)** Agarose gel analysis of representative qRT-PCR products confirms absence of a specific amplification product all samples from 5-wk old mice. LN, lymph node; TEB, terminal end bud. Scale bars 100 µm.

Figure S2.

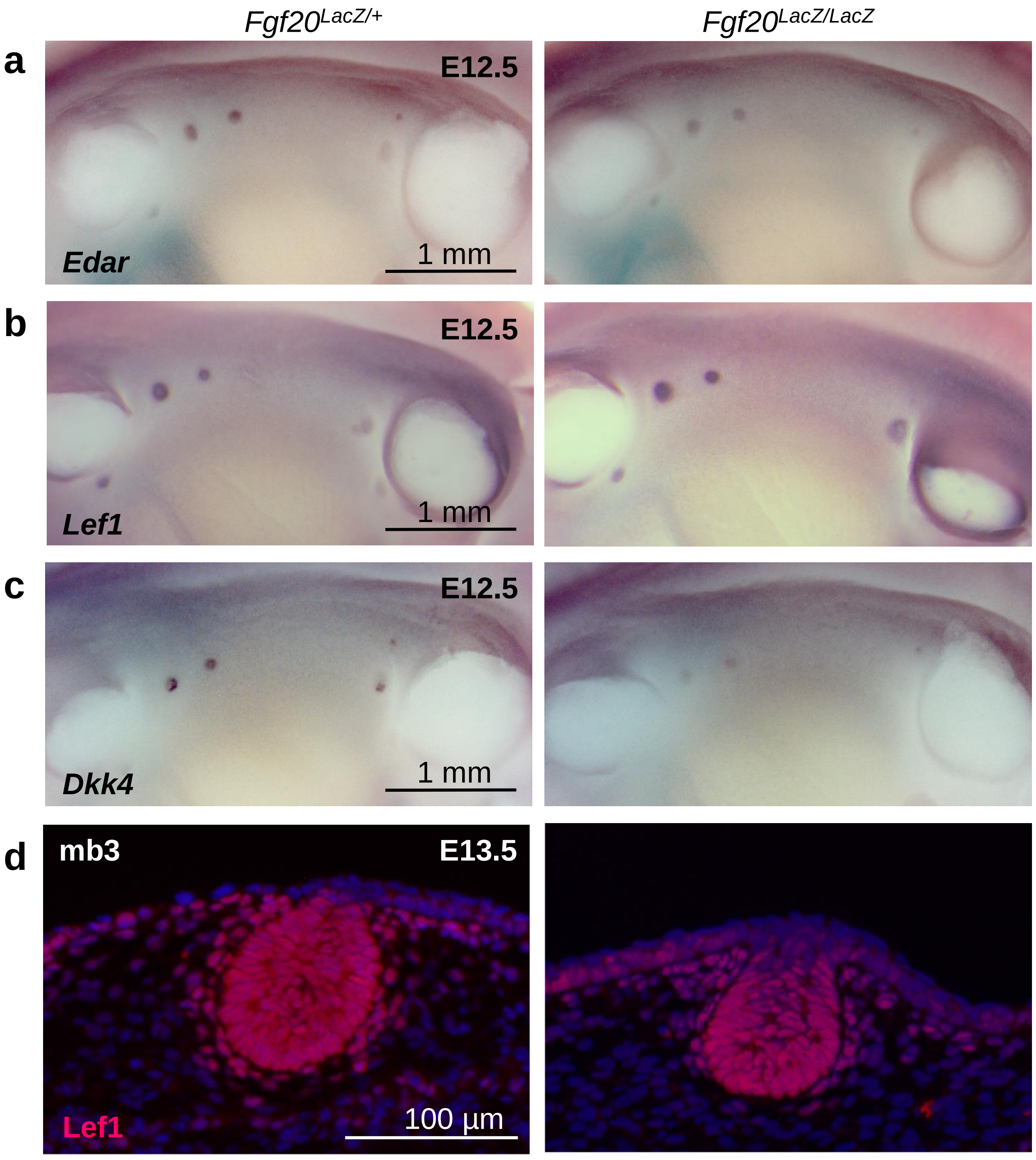

Supplementary Figure S2. Expression of mammary bud markers in *Fgf20*$^{LacZ/+}$ and *Fgf20*$^{LacZ/LacZ}$ embryos. **(a-c)** Whole mount in situ hybridization with probes specific for *Edar* (n=6 for both genotypes), *Lef1* (n=4), and *Dkk4* (n=7) at E12.5, and **(d)** Lef1 protein expression in mammary bud 3 (n=3) at E13.5, nuclei are stained blue.

Figure S3.

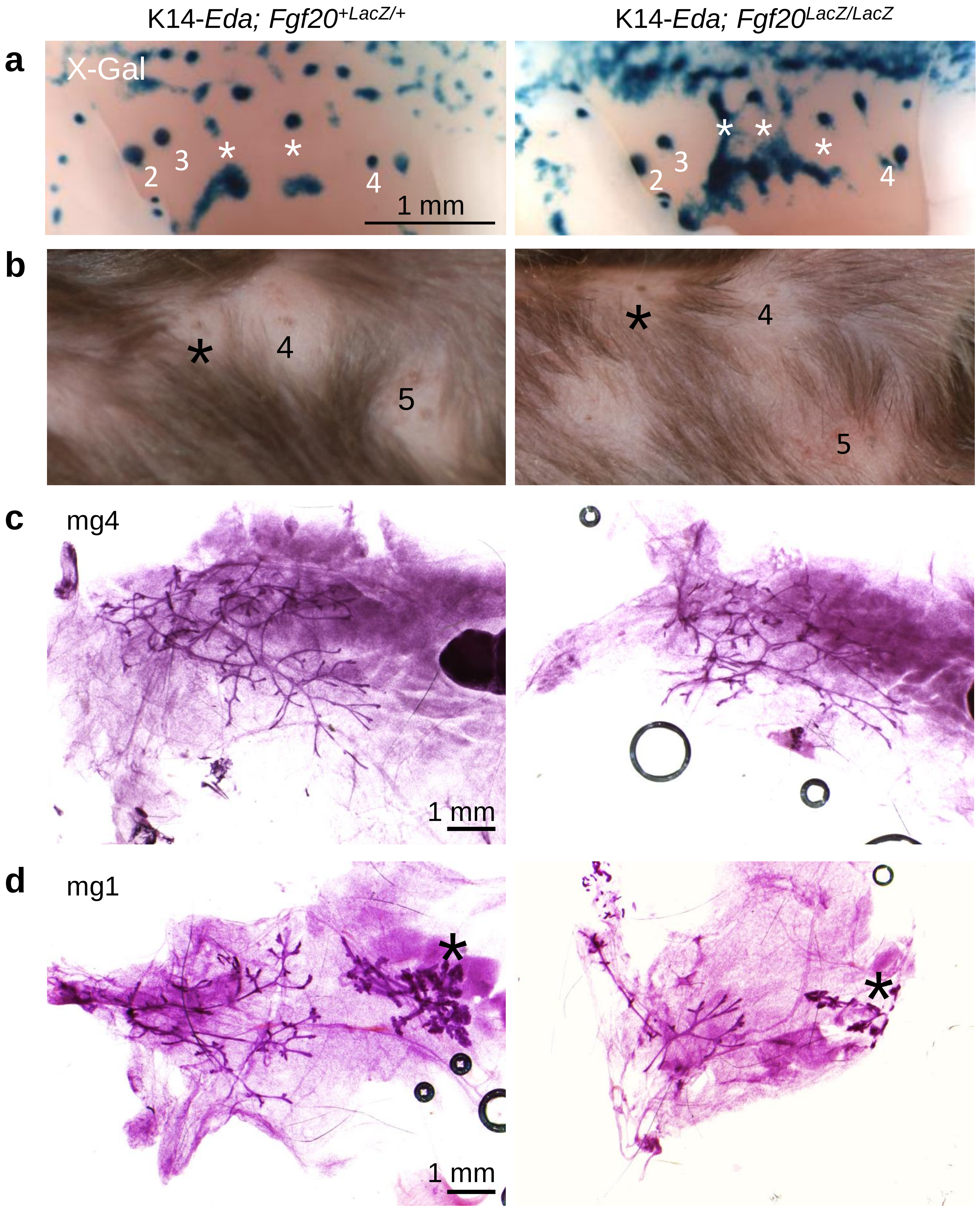

Supplementary Figure S3. Development of supernumerary mammary glands in K14-*Eda;Fgf20*$^{LacZ/+}$ and K14-*Eda;Fgf20*$^{LacZ/LacZ}$ mice. (**a**) X-Gal staining of K14-*Eda;Fgf20*$^{LacZ/+}$ and K14-*Eda;Fgf20*$^{+LacZ/LacZ}$ female embryos at ~E14.0 revealed development of supernumerary mammary buds (stars) also in the K14-*Eda; Fgf20*$^{+LacZ/LacZ}$ embryos. (**b**) Supernumerary nipples (stars) were found in the milk line of postnatal (7 week old) K14-*Eda;Fgf20*$^{LacZ/+}$ and K14-*Eda;Fgf20*$^{LacZ/LacZ}$ females. (**c,d**) Presence of mammary glands in K14-*Eda;Fgf20*$^{LacZ/+}$ and K14-*Eda;Fgf20*$^{LacZ/LacZ}$ males was studied at different ages (3, 4, 6 and 22 weeks) by Carmine alum staining. This revealed that ductal trees were present in both genotypes. Representative figures of mammary gland 4 (**c**) and 1 (**d**), including supernumerary mammary gland in the neck (stars), from 4-week old males are shown.

Figure S4.

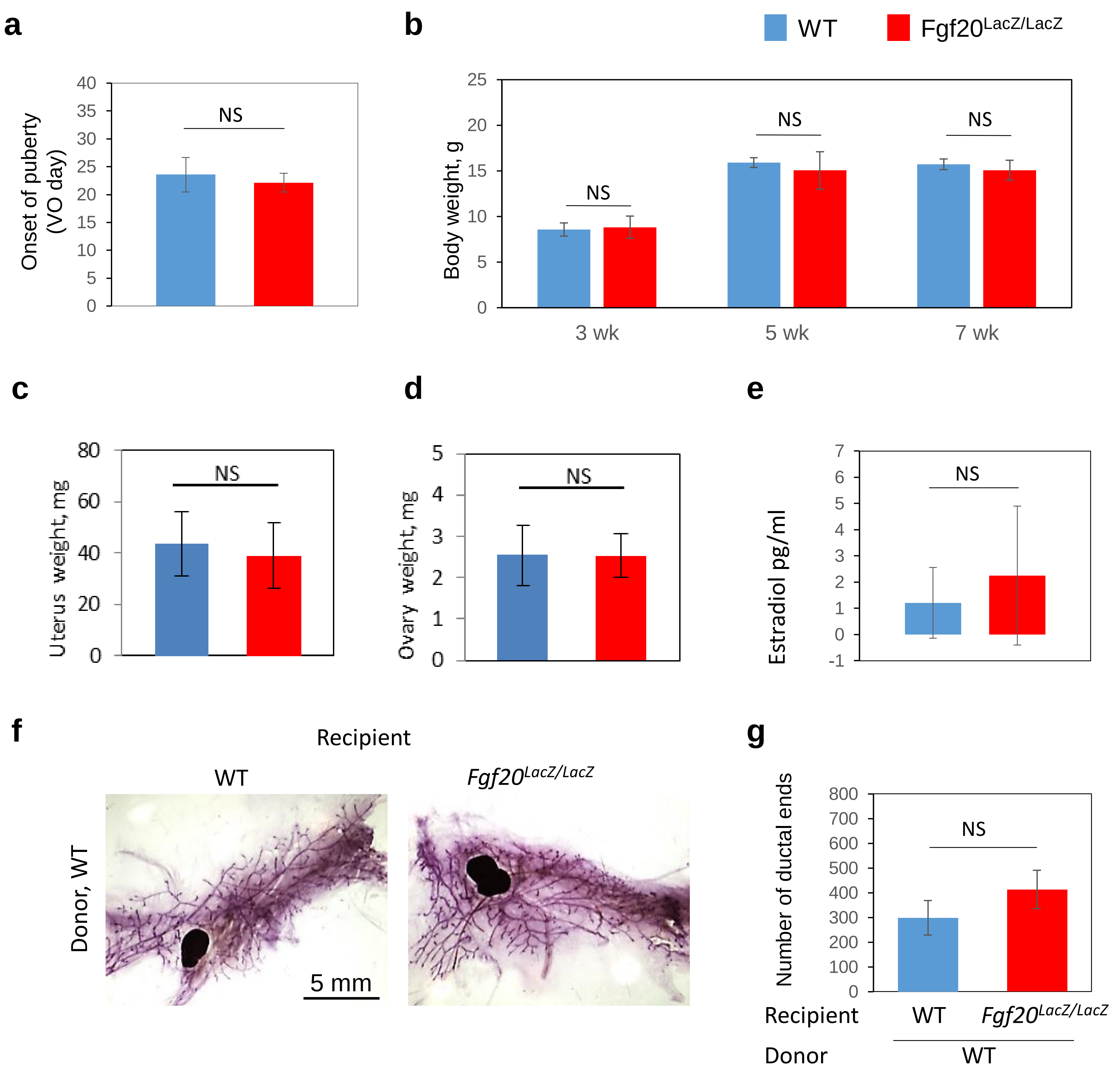

Supplementary Figure S4. Analysis of various parameters of puberty in *Fgf20* deficient females. (**a**) Day of the vaginal opening (VO) in WT (n=10) and *Fgf20*$^{LacZ/LacZ}$ (n=11) females did not differ between the genotypes. (**b**) Body weight at 3 weeks (WT, n=15; *Fgf20*$^{LacZ/LacZ}$, n=9), 5 weeks (WT, n=6; *Fgf20*$^{LacZ/LacZ}$, n=10), and 7 weeks (WT, n=9; *Fgf20*$^{LacZ/LacZ}$, n=12) of age displayed no significant difference (p_{3wk}=0.588; p_{5wk}=0.239; p_{7wk}=0.096). (**c**) Uterus (WT, n=6; *Fgf20*$^{LacZ/LacZ}$, n=8) and (**d**) ovary (WT, $n_{ovaries}$=13; *Fgf20*$^{LacZ/LacZ}$, n=16) weight at 7 weeks of age did not show significant differences between WT and *Fgf20*$^{LacZ/LacZ}$ females (p_{ovary}=0.915; p_{uterus}=0.499). (**e**) Serum estradiol levels in 7-week-old WT (n=8) and *Fgf20*$^{LacZ/LacZ}$ mice (n=9) were measured in diestrus and they did not significantly differ between the genotypes (p=0.315). (**f**) Representative figures of ductal trees in the mammary fat pads of 8-9-week-old WT and *Fgf20*$^{LacZ/LacZ}$ females five weeks after being transplanted with 1 mm^3 pieces of ductal epithelium of 12-13 week old WT mammary glands. (**g**) Quantification of ductal ends in the WT (n=5) and *Fgf20*$^{LacZ/LacZ}$ (n=4) transplant recipients (p=0.052). Values represent mean ±SD. NS, not significant (p>0.05).

Figure S5.

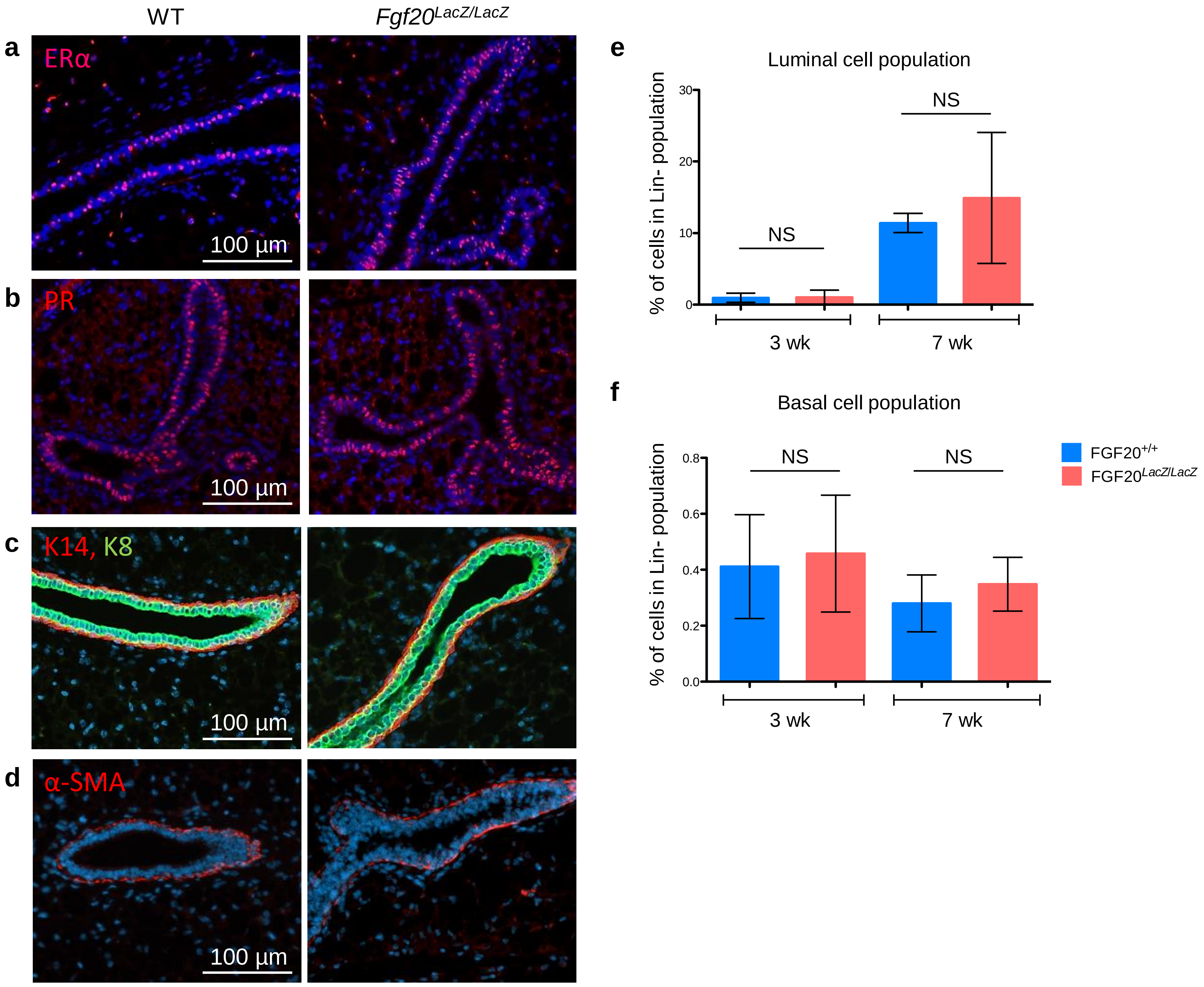

Supplementary Figure S5. Normal duct architecture in the *Fgf20* deficient mammary glands. (**a-d**) Immunohistochemical stainings of ERα, PR, K14, K8, and α-SMA in mammary ducts of WT and $Fgf20^{LacZ/LacZ}$ mice at 7 weeks of age did not show any obvious differences. Nuclei were stained blue. (**e-f**) Percentage of luminal ($CD29^{lo}CD24^{+}$) (**e**) and basal ($CD29^{hi}$ $CD24^{+}$) cells (**f**) within the lineage-negative cells in the mammary glands of 3-week-old (n_{mg}=11) and 7-week-old WT (n_{mg}=6) and $Fgf20^{LacZ/LacZ}$ mice (n_{mg}=7). Values represent mean ±SD. No significant differences between WT and $Fgf20^{LacZ/LacZ}$ mice were found at 3 weeks ($p_{luminal}$=0.3740; p_{basal}=0.2371) or 7 weeks ($p_{luminal}$=0.8179; p_{basal}=0.5848). mg, mammary gland. NS, non significant (p>0.05).
